# Baseline susceptibility of an A1 quarantine pest - the South American tomato pinworm *Tuta absoluta* (Lepidoptera: Gelechiidae) to insecticides: past incidents and future probabilities in line to implementing successful pest management

**DOI:** 10.3389/fpls.2024.1404250

**Published:** 2024-08-26

**Authors:** Chandra Mohan Muthu Lakshmi Bavithra, Marimuthu Murugan, Venkatasamy Balasubramani, Sankarasubramanian Harish, Kolanchi Prakash

**Affiliations:** ^1^ Department of Agricultural Entomology, Tamil Nadu Agricultural University, Coimbatore, India; ^2^ Controller of Examinations, Tamil Nadu Agricultural University, Coimbatore, India; ^3^ Department of Plant Pathology, Tamil Nadu Agricultural University, Coimbatore, India

**Keywords:** tomato pinworm, baseline susceptibility, insecticide resistance, resistance ratio, insecticide resistance management

## Abstract

Tomato is a widely cultivated crop significant for its economic and nutritional benefits. The South American tomato pinworm, *Tuta absoluta*, originated in Peru South America and has invaded many nations, causing up to 100% yield loss in tomatoes. The pest was classified as a quarantine pest by the European Plant Protection Organization, before invading the Spain region. Later, this quarantine pest also invaded other regions of Europe, Africa and Asian countries. Invasive insect pests cause global economic losses of 70 billion dollars annually. Among the several management measures suggested against pests, insecticides are the primary method in practice among growers due to significant results, easier operations, and other crucial advantages. Anyhow, repeated application of insecticides has caused the pest to evolve resistance against most of the insecticides in vogue, resulting in a chain of events like management failures, using increased doses of insecticides, intensified chemical residues in the food chain, and irreparable environmental contamination. Major insecticides globally used to control *T. absoluta* belong to organophosphates, synthetic pyrethroids, neonicotinoids, diamides, avermectins, spinosyns, and oxadizines. Understanding the baseline susceptibility of pests to insecticides helps for better pest management options and is the same for *T. absoluta* populations to insecticides. The current review paper discusses the *T. absoluta* distribution, biology, spread, host range, baseline insecticide susceptibility, global insecticide resistance status, and possible management inputs based on our understanding of insecticide susceptibility. The pest can be managed with integrated insecticide resistance management including molecular approaches.

## Introduction

Tomato (*Solanum lycopersicum* Mill.), which belongs to the family Solanaceae and originated from the western coastal part of South America, is a widely cultivated worldwide crop due to its high nutritional value (Peralta et al., 2008). According to the Food and Agricultural Organization (FAO), total world tomato production for processing and fresh consumption in 2021 was about 189.1 million metric tons (FAO, 2021). China is the leading producer of tomatoes, accounting for 67.3 million metric tons, followed by India, Turkey, USA, and Italy. Various biotic and abiotic elements interfere with the plant’s ability to grow. Among the biotic factors, the insect pests such as Tomato fruit borer *Helicoverpa armigera* (Hubner) (Lepidoptera: Noctuidae), leaf miner *Liriomyza trifolii* (Burgess) (Diptera: Agromyzidae), Tomato Pinworm, *Tuta absoluta* Meyrick (Lepidoptera: Gelechiidae) and whitefly, *Bemisia tabaci* (Gennadius) (Hemiptera: Aleyrodidae), affect the overall tomato production (Pandey et al., 2017; Wakil et al., 2018). Among all the insect pests, the invasive pest of tomato, the South American pinworm, *T. absoluta* is a major concern for tomato production. Originally endemic to South America, *T. absoluta* has rapidly expanded its geographic range, establishing itself as a major pest in Africa, Europe, and Asia. *T. absoluta* infests young plants by burrowing into the buds and stems. As the foliage grows, larvae destroy the leaves by mining and reduce their ability to perform photosynthesis. *T. absoluta* is a challenging target for pesticide sprays due to the larvae-feeding nature and structure of the plant (Guedes and Siqueira, 2012; Biondi et al., 2018). Effective control of *T. absoluta* has predominantly relied on chemical insecticides to avoid outbreaks. Factors such as applying insecticides to control pests that interfere with production are causing severe threats like insecticide resistance and pest resurgence. Historical incidents of resistance to key insecticides, such as organophosphates, pyrethroids, and neonicotinoids, highlight the urgent need for integrated pest management (IPM) strategies that reduce reliance on chemical controls and mitigate resistance development (Campos et al., 2014; Roditakis et al., 2015). The invasive nature and high reproductive potential, coupled with its ability to develop insecticide resistance, pose significant challenges to effective pest management (Desneux et al., 2010; Guedes and Picanço, 2012). Insects develop insecticide resistance through different resistance mechanisms of which metabolic resistance and target site alterations are the most common mechanisms of resistance in *T. absoluta* (Konuş, 2014). This review aims to provide a comprehensive analysis of the baseline susceptibility of *T. absoluta* to various insecticides, documenting past incidents of resistance and evaluating future probabilities of resistance development. It also focuses on the biology, origin, spread, host range and insecticidal usage pattern of *T. absoluta.* This involves not only understanding the mechanisms and factors contributing to resistance but also exploring alternative control methods and the integration of novel technologies.

## Biology of *Tuta absoluta*

The gravid females lay 260 to 300 eggs, which are small, single, cylindrical oysters of a creamy yellow color of 0.35 mm long. The eggs are laid mainly on the underside of leaves, growing buds, stems, and calyx of unripe fruit or alongside the rachis (Salama et al., 2014). Freshly emerged larvae are light yellow or greenish and only 0.5 mm long. The larval stage duration ranges from 10 to 15 days. The late instar larvae consist of a characteristic dark band posterior to the head region. Larvae form mines by feeding only on mesophyll tissues of the leaf, and the epidermis is left intact (EPPO, 2005). They also feed on fruits, stalks, and apical buds, forming mines and galleries (Biondi et al., 2018). The mature larvae pupate in soil or silken cocoons in the leaves or mines (Batalla-Carrera et al., 2010). The pupal stage lasts 9 to 11 days (Ballal et al., 2016). Adults are small, about 5-7 mm long, with filiform antennae, silverish-grey scales, and distinct black spots on forewings. The labial palps are recurved and well-developed (Berxolli and Shahini, 2017). The life span of males is 6 to 7 days, and for females, 10 to 15 days. The life cycle completes within 30–35 days and develops 10-12 generations/year (Desneux et al., 2010).

The life cycle of *T. absoluta* is one of the main elements influencing insecticide resistance development. High reproduction capacity and short life cycle pose a higher risk of developing resistance to insecticides. Larvae and pupae are carried from one place to another through infested fruits which allows the mixing of individuals exposed to different insecticides. Selection pressure of insecticides also frequently arises during the development stages of the pest (Sudo et al., 2018).

### Origin and spread

The tomato pinworm, *Tuta absoluta* Meyrick (Lepidoptera: Gelechiidae) is an invasive pest native to South America. *T. absoluta* was first reported in Huancayo, Peru, in 1917 and it was later found in eastern Spain in the year 2006. The pest has spread worldwide in South and Central America as well as Europe, Africa, and the Middle East (Desneux et al., 2010, Desneux et al., 2011; Tropea Garzia et al., 2012; Zappalà et al., 2013; Brévault et al., 2014). It has widely spread over North Africa (Algeria, Morocco and Tunisia), Europe (France, Italy, Netherlands, United Kingdom, Albania, Portugal, Malta and Switzerland) and Asia (United Arab Emirates, Yemen, India Israel, Iraq and Qatar). *T. absoluta* reported for the first time in various countries has been described in **Supplementary Table S1**.

### Host range of *T. absoluta*

Though tomato is the primary host of *T. absoluta* (EPPO, 2005), it also infests several other solanaceous and non-solanaceous cultivated crops and weeds. According to reports, it can infest solanaceous crops such as potato (*S. tuberosum* L.), eggplant (*S. melongena* L.), pepper (*Capsicum annum* L.), African aubergine (*S. aethiopicum* L.), and tobacco (*Nicotiana tabacum* L.) and solanaceous weeds like *Datura stramonium* L., and *N. glauca* G. It also feeds on non-solanaceous plants like green beans (*Phaseolus vulgaris* L.), and *Malva* spp (EPPO, 2005; Mohamed et al., 2015).

According to the European and Mediterranean Plant Protection Organization (EPPO), the host plants of pests have been differentiated into seven categories: major host, host, alternate, wild/weed, experimental, doubtful host, and non-host. In the case of *T. absoluta*, the major host is tomato. The other wild/weed and experimental hosts are listed in **Table 1**.

**Table 1:** List of cultivated, wild/weed, and experimental host plants that are utilized by *T. absoluta* to complete their life cycle and pass on to the next generation.

Common name of plants	Scientific name	Family	References
Cultivated plant species			
Beetroot	*Beta vulgaris*	Amaranthaceae	(EPPO, 2024)
Watermelon	*Citrullus lanatus*	Cucurbitaceae	(EPPO, 2024)
Nutmeg	*Jatropha curcas*	Euphorbiaceae	(EPPO, 2024)
Lucerne	*Medicago sativa*	Fabaceae	(EPPO, 2024)
Common bean	*Phaseolus vulgaris*	Fabaceae	(Idriss et al., 2020)
African eggplant	*Solanum aethiopicum*	Solanaceae	(Sawadogo et al., 2022)
Eggplant or Aubergine or Guinea squash	*Solanum melongena*	Solanaceae	(Idriss et al., 2020)
Pepino	*Solanum muricatum*	Solanaceae	(EPPO, 2024)
Potato	*Solanum tuberosum*	Solanaceae	(EPPO, 2024)
Spinach	*Spinacia oleracea*	Amaranthaceae	(EPPO, 2024)
Wild/weed plants			
Spiny amaranth	*Amaranthus spinosus*	Amaranthaceae	(EPPO, 2024)
Good-king-Henry	*Blitum bonus-henricus*	Amaranthaceae	(EPPO, 2024)
Fierce thornapple	*Datura ferox*	Solanaceae	(EPPO, 2024)
Jimson weed	*Datura stramonium*	Solanaceae	(EPPO, 2024)
Boxthorn	*Lycium chilense*	Solanaceae	(EPPO, 2024)
Tree tobacco	*Nicotiana glauca*	Solanaceae	(EPPO, 2024)
Red goosefoot	*Oxybasis rubra*	Amaranthaceae	(EPPO, 2024)
Jdadgo	*Solanum coagulans*	Solanaceae	(Idriss et al., 2020)
Silverleaf nightshade	*Solanum elaeagnifolium*	Solanaceae	(EPPO, 2024)
Hairy tomato	*Solanum habrochaites*	Solanaceae	(Aslan et al., 2022)
Lyreleaf nightshade	*Solanum lyratum*	Solanaceae	(EPPO, 2024)
Black nightshade	*Solanum nigrum*	Solanaceae	(Arno et al., 2019)
Hairy nightshade	*Solanum sarrachoides*	Solanaceae	(Arno et al., 2019)
Rough cocklebur	*Xanthium strumarium*	Asteraceae	(EPPO, 2024)
Experimental host plants			
Wild tomato	*Solanum arcanum*	Solanaceae	(Aslan et al., 2022)
Peruvian nightshade	*Solanum peruvianum*	Solanaceae	(Aslan et al., 2022)
Currant tomato	*Solanum pimpinellifolium*	Solanaceae	(Aslan et al., 2022)

### Insecticides used against *T. absoluta* and their usage pattern

Few insecticides of organophosphates and pyrethroids were initially available for *T. absoluta* control in the early 1960s and until the 1980s (Siqueira et al., 2000; Salazar and Araya, 2001; Lietti et al., 2005). Organophosphates quickly proved less effective, and pyrethroids were subsequently combined with cartap (nereistoxin analog) and abamectin (avermectin) as a replacement (Siqueira et al., 2000; Siqueira et al., 2001; Guedes and Siqueira, 2012). Indoxacarb, diflubenzuron, teflubenzuron, and triflumuron were used during the late 1990s and early 2000s (Silva et al., 2011; Guedes and Picanço, 2012). The pyrroles, spinosyns, and diamides (chlorantraniliprole and flubendiamide) were novel chemical classes used to control *T. absoluta* (Silva et al., 2011; Gontijo et al., 2013; Silva et al., 2016a, Silva et al., 2016b). The major pesticides used for organic tomato production were azadirachtin, spinosad and *Bacillus thuringiensis* toxins (Bt toxins) (Silva et al., 2011; Biondi et al., 2018).

### Registered insecticides and their mode of action

The list of insecticides registered for the control of *T. absoluta* and their corresponding mode of action with the Insecticide Resistance Action Committee (IRAC) classification is represented in **Table 2** (IRAC, 2020). IRAC, a technical working group under CropLife International, coordinates efforts within the crop protection industry to prevent or delay the development of resistance in insect, mite, and tick pests (Nauen et al., 2019).

**Table 2:** Global list of insecticides registered for use in the control of *T. absoluta*.

Insecticide class	Name of compounds	IRAC group	Mode of action
Organophosphates	Chlorpyrifos, Methamidophos	1B	Acetylcholinesterase (AChE) inhibitors
Pyrethroids	Bifenthrin, Cyfluthrin, beta Cyfluthrin, gamma-Cyhalothrin, Cypermethrin, alpha-Cypermethrin, beta-Cypermethrin, theta cypermethrin, zeta-Cypermethrin, Deltamethrin, Esfenvalerate, Etofenprox, Fenpropathrin, tau-Fluvalinate, Permethrin	3A	Sodium channel modulators
Spinosyns	Spinetoram, Spinosad	5	Nicotinic acetylcholine receptor (nAChR) allosteric modulators – Site I
Avermectins	Abamectin, Emamectin benzoate	6	Glutamate-gated chloride channel (GluCl) allosteric modulators
Pyrroles	Chlorfenapyr	13	Uncouplers of oxidative phosphorylation via disruption of the proton gradient
Nereistoxin analogues	Cartap hydrochloride	14	Nicotinic acetylcholine receptor (nAChR) channel blockers
Benzoylureas	Diflubenzuron, Flucycloxuron, Lufenuron, Novaluron, Noviflumuron, Teflubenzuron, Triflumuron	15	Inhibitors of chitin biosynthesis affecting Chitin synthetase 1 (CHS1)
Diacylhydrazines	Chromafenozide, Methoxyfenozide, Tebufenozide	18	Ecdysone receptor agonists
Oxadiazines	Indoxacarb	22A	Voltage-dependent sodium channel blockers
Semicarbazones	Metaflumizone	22B	Voltage-dependent sodium channel blockers
Diamides	Chlorantraniliprole, Cyantraniliprole, Flubendiamide,	28	Ryanodine receptor modulators

### Insecticide resistance in *T. absoluta*

**Figure 1:**
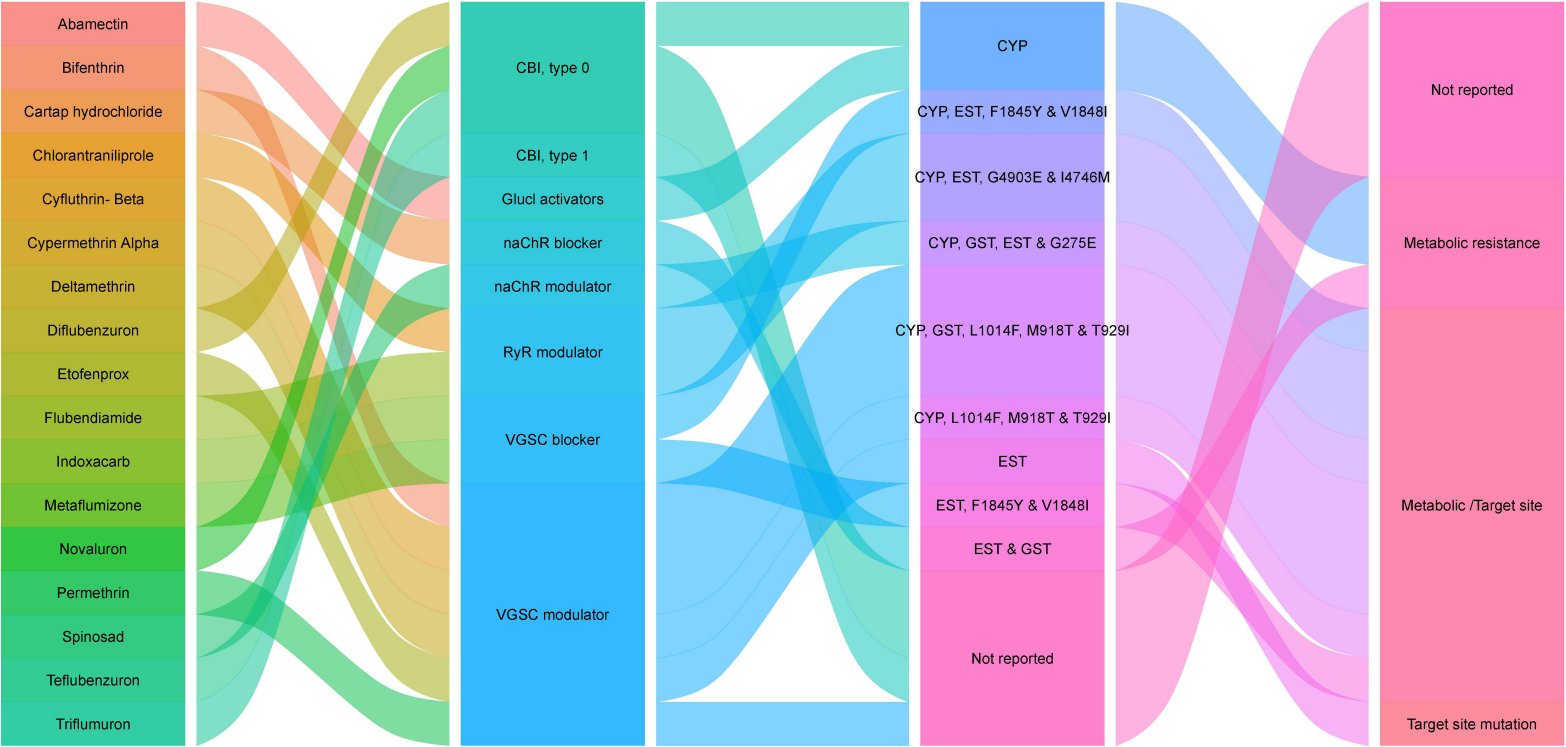
Sankey map representing insecticides and their resistance mechanisms against *T. absoluta*.

Arthropod Pesticide Resistance Database (APRD) is a web-based resistance case entry system that acts as a resource for arthropod resistance information and offers a platform for scale-appropriate, real-time reporting of the global present status of arthropod resistance. The information provided by APRD is based on the data submitted by the registered users and approved by a peer-review panel of resistance experts. According to APRD, 59 insecticide-resistance cases have been reported on *T. absoluta*. The wholesome information related to the insecticide resistance of *T. absoluta* collected from APRD (2024) is depicted in **Table 3**. The insecticides for which *T. absoluta* has become resistant, the corresponding mode of action and their resistance mechanism are depicted in **Figure 1**.

**Table 3:** Details of insecticide resistance prevalent in *T. absoluta* populations in different countries (From APRD website).

Insecticide	MOA	No. of cases reported	Year of report	Type of resistance	Mechanism of resistance	Location	Reference
Abamectin	Chloride channel activators	6	2001	Field Evolved Resistance	Metabolic resistance	Brazil	(Siqueira et al., 2001)
Bifenthrin	Sodium channel modulators	1	2011	Lab selected after field-evolved resistance	Target site mutation	Brazil	(Silva et al., 2011)
Cartap	Nicotinic Acetylcholine receptor agonists/antagonists	4	2000	Field Evolved Resistance (3 cases) Lab selected (1 case)	Metabolic resistance	Brazil	(Siqueira et al., 2001)
Chlorantraniliprole	Ryanodine receptor modulators	4	2019	Lab selected after field-evolved resistance (1 Case) Field Evolved Resistance (3 Cases)	Metabolic resistance and Target site mutation	United Kingdom	(Grant et al., 2019)
			2023			Spain	(Grant et al., 2023)
Cyfluthrin-Beta	Sodium channel modulators	1	2014	Field Evolved Resistance	Metabolic resistance and Target site mutation	Brazil	(Barros et al., 2014)
Cypermethrin-Alpha	Sodium channel modulators	1	2015	Field Evolved Resistance	Metabolic resistance and Target site mutation	Brazil	(Silva et al., 2015)
Deltamethrin	Sodium channel modulators	2	2005	Field Evolved Resistance	Metabolic resistance and Target site mutation	Argentina	(Lietti et al., 2005)
Diflubenzuron	Inhibitors of chitin biosynthesis, type 0	2	2011	Lab selected after field-evolved resistance	Metabolic resistance	Brazil	(Silva et al., 2011)
Etofenprox	Sodium channel modulators	1	2014	Field Evolved Resistance	–	Brazil	(Barros et al., 2014)
Flubendiamide	Ryanodine modulators	1	2017	Lab selected	Metabolic resistance and Target site mutation	Greece	(Douris et al., 2017)
Indoxacarb	Voltage-dependent sodium channel blocker	6	2011	Lab selected after field-evolved resistance (3 cases)	Metabolic resistance and Target site mutation	Brazil	(Silva et al., 2011)
			2023	Field Evolved Resistance (3 cases)		Iran	Aboutalebian-Soureshjani et al., 2023)
Metaflumizone	Voltage-dependent sodium channel blocker	3	2016	Field Evolved Resistance	Metabolic resistance and Target site mutation	Brazil	(Silva et al., 2016b)
Novaluron	Inhibitors of chitin biosynthesis, type 0	1	2021	Field Evolved Resistance	–	Brazil	(Silva et al., 2021)
Permethrin	Sodium channel modulators	2	2014	Field Evolved Resistance	Metabolic resistance and Target site mutation	Brazil	(Barros et al., 2014)
			2011	Lab selected after field-evolved resistance	Metabolic resistance and Target site mutation		(Silva et al., 2011)
Spinosad	Nicotinic Acetylcholine receptor agonists	16	2012, 2015, 2016, 2019	Field Evolved Resistance	Metabolic resistance and Target site mutation	Brazil, UK, Chile	(Campos et al., 2015; Grant et al., 2019; Silva et al., 2016a; Reyes et al., 2012)
				Lab selected after field-evolved resistance	Metabolic resistance and Target site mutation		
Teflubenzuron	Inhibitors of chitin biosynthesis, type 0	3	2011	Lab selected after field-evolved resistance	–	Brazil	(Silva et al., 2011)
Triflumuron	Inhibitors of chitin biosynthesis, type 0	3	2011	Lab selected after field-evolved resistance	–	Brazil	(Silva et al., 2011)

### Global resistance status

*T. absoluta* field populations were known to be highly resistant in Latin America, particularly to organophosphates (MoA [Mode of Action] group 1B), pyrethroids (MoA group 3A), chloride channel activators (MoA group 6) and benzoylureas (MoA group 15). The more common resistance mechanism was metabolic resistance which increases the activity of detoxification enzymes such as Cytochrome P 450 oxidase, Glutathione S transferase and esterase. In addition, the causes of resistance could involve multiple genes and be polyfactorial. Resistance to newer insecticide classes released after 2000 has also grown in Latin America (Silva et al., 2011). In Chile, the usage of pyrethroids and organophosphates led to resistance for the first time, later both pyrethroids and organophosphate resistance was also shown in Brazil and Argentina populations (Siqueira et al., 2000; Salazar and Araya, 2001; Lietti et al., 2005). After *T. absoluta* invaded from South America to Europe, pyrethroid resistance also became widespread (Siqueira et al., 2000; Silva et al., 2011, Silva et al., 2015). Then *T. absoluta* attained low-to-moderate levels of resistance to cartap and abamectin with a gradual increase in resistance level (Siqueira et al., 2000; Siqueira et al., 2001; Silva et al., 2016b). Later, indoxacarb was found to develop low-to-moderate level resistance against *T. absoluta* (Silva et al., 2011, Silva et al., 2016b). In the mid-2000s, high resistance of chitin biosynthesis inhibitors was detected due to over-usage (Silva et al., 2011). Chitin biosynthesis inhibitors became less effective due to increased resistance, and then, spinosad use increased in place of chitin biosynthesis inhibitors, finally leading to varying levels of spinosad resistance (Reyes et al., 2012; Campos et al., 2014, Campos et al., 2015). To overcome the inefficiency of all other insecticides to which *T. absoluta* is resistant, a new chemical class of insecticide diamides was applied for tomato pinworm control (Nauen, 2006), but after extensive usage for a few years, the resistance was detected for these compounds also (Campos et al., 2015; Roditakis et al., 2015; Silva et al., 2016a; Roditakis et al., 2017; Silva et al., 2019).

### Baseline susceptibility

Reliable baseline data on the target pest’s susceptibility to the toxicant is a crucial aspect in managing the usage of insecticides and acaricides. According to IRAC, data from a pest strain that has never been selected against the toxicant or from toxicants that exhibit cross-resistance at a similar site of action are known as baseline data.

For baseline susceptibility and resistance monitoring studies, bioassay on the test insecticides should be conducted to find the medial lethal dose or concentration (LD_50_ or LC_50_) values and to determine the amount of active ingredient required to kill 50% of the population. Among the different bioassay methods, IRAC method no 22 was recommended for *T. absoluta*, and leaf dip bioassay is performed preferably on second-instar larvae. The susceptible population without insecticide exposure is considered as a control for comparison (IRAC, 2014). Details of the baseline susceptibility of different insecticides of different populations belonging to various countries are listed in **Supplementary Table S3**. Additional information including LC50 and RR has been described in **Supplementary Table S2**.

### Resistance ratio

The resistance ratio is used to monitor the evolution of resistance in field populations compared to a susceptible population. RR is calculated by dividing the LC50 of the field population by the LC50 of a susceptible population. Resistance level classification: susceptible (<3-fold RR), minor resistance (3 to 5-fold RR), low resistance (5 to 10-fold RR), intermediate resistance (10 to 40-fold RR), high resistance (40 to 160-fold RR), and extremely high resistance (RR >160-fold) (Jin et al., 2016).

## Continent/countrywide status in baseline susceptibility

### Argentina

Initially, organophosphates were the only insecticides used against *T. absoluta* in Argentina. However, during the 1970s, pyrethroids progressively replaced organophosphates. Cartap, developed in the early 1980s showed outstanding efficacy, alternated with pyrethroids and thiocyclam hydrogen oxalate insecticides. Insecticides with novel sites of action were developed in the 1990s, including spinosad, tebufenozide, acylurea insect growth regulators, abamectin, and chlorfenapyr (Lietti et al., 2005).

Based on RR values, the Argentina population has shown a high level (RR > 68.38) of resistance to deltamethrin, which is a synthetic pyrethroid. In contrast, the avermectin abamectin showed minor resistance (RR: 3.49) and organophosphates methamidophos was susceptible (RR: 0.86) (Lietti et al., 2005).

### Brazil

Repeated application of insecticides during a single tomato cultivation period has led to the development of resistant populations among Tuta. In Brazil, only permethrin and cartap were used over an extended period to control *T. absoluta* (Souza and Reis, 1986), and during the early 1990s, abamectin and methamidophos were under use.

Between 1997 and 1998, the Brazilian population of *T. absoluta* exhibited varying levels of resistance to different insecticides. Methamidophos showed a minor level of resistance (RR: 4.22), abamectin displayed a low level of resistance (RR: 9.37), and permethrin ranged from minor to low-level resistance (RR: 3.31 to 6.61). However, for cartap, an intermediate resistance level (RR: 21.9) was observed (Siquiera et al., 2000).

Similarly, in 2010-2011, Brazilian populations showed a wider range of resistance to different insecticides. Diamides such as chlorantraniliprole, cyantraniliprole, and flubendiamide showed resistance ratios (RR) indicating minor to low resistance (RR: 3.22 to 9.33), minor resistance (RR: 3.36), and susceptibility (RR: 2.45), respectively (Campos et al., 2014). Additionally, these populations demonstrate varying resistance levels to other insecticides: indoxacarb (RR: 3.3) and chlorfenapyr (RR: 4.6) with minor resistance, abamectin (RR: 3 to 6.2), cartap (RR: 3.4 to 6.4) shown minor to low-level resistance and metaflumizone (RR: 7.4 to 21.2) exhibited low to intermediate levels of resistance (Silva et al., 2016a).

Between 2010 and 2015, another Brazilian population studied exclusively for resistance to diamides such as chlorantraniliprole, cyantraniliprole, and flubendiamide showed varied resistance levels. The resistance levels for flubendiamide varied significantly, ranging from low to extremely high (RR: 7.95 to 80413). In contrast, chlorantraniliprole and cyantraniliprole exhibited high to extremely high resistance levels with RR 44.55 to 288995-fold and 78.34 to 18423-fold, respectively (Silva et al., 2016b).

Overall, the various insecticides used in Brazil against *T. absoluta* from 1997 to 2015 have revealed that RR has varied from susceptible to extremely high levels of resistance. The diamides, in particular, which have exhibited extremely high resistance levels compared to other insecticides, necessitate effective insecticide resistance management.

### Europe

The most extensively used insecticides to control *T. absoluta* include diamides, avermectins, spinosyns, and oxadiazines (Sparks and Nauen, 2015). Chlorantraniliprole is currently the only diamide insecticide registered to control *T. absoluta* in Europe. In the avermectin class, abamectin and emamectin benzoate were registered. Spinosad and indoxacarb were registered to control *T. absoluta* belonging to the classes spinosyns and oxadizines, respectively (Roditakis et al., 2018).

Insecticide resistance studies was conducted on European populations of *T. absoluta* in 2009 to 2011 against diamide, chlorantraniliprole and oxadizine, indoxacarb. The RR fold was 6-fold for chlorantraniliprole which is a low-level resistance. In contrast, indoxacarb (RR: 4 to 12) exhibited a minor to low-level resistance (Roditakis et al., 2013).

A four-year survey from 2012 to 2016 on baseline susceptibility of *T. absoluta* in the European populations using insecticides with different modes of action such as chlorantraniliprole, emamectin benzoate, spinosad, and indoxacarb. RR value of chlorantraniliprole was found to be maximum of 22,573-fold which is extremely high level of resistance whereas, intermediate level of resistance was noticed for emamectin benzoate (RR: 35). But the population had high level resistance against indoxacarb and intermediate resistance for Spinosad with RR values 91 and 33, respectively (Roditakis et al., 2018).

The drastic variation in the resistance status of chlorantraniliprole was found with resistance from 12-fold to 22573-fold and the fold change was observed within a period of 3 years.

### Turkey

Some key insecticides sprayed in Turkey for *T. absoluta* control were chlorantraniliprole, metaflumizone, indoxacarb, spinosad, and abamectin. During 2011-2012, *T. absoluta* field samples of Turkey were susceptible to abamectin (RR: 3.03) which act as Glutamate-gated chloride channel (GluCl) allosteric modulators (Konuş, 2014. In contrast, the Turkey population tested against Voltage-dependent sodium channel blockers, metaflumizone (RR: 3.79) and indoxacarb (RR: 8.02) shown minor and low level of resistance, respectively (Yalcin et al., 2015).

### Iran

Indoxacarb toxicity studied against six field populations of *T. absoluta* collected from different provinces of Iran (Benoot-e Bala, Ardabil, Safiabad, Parsabad Moghan, Mohammad Shahr, and Ziba Shahr) in the year 2020-2021 exhibited a resistance of minor to intermediate level (4.22-25.83) (Taleh et al., 2023). Similarly, other populations tested with abamectin also showed minor to intermediate resistance level (RR: 4.28-25.25) (Azizi and Khajehali, 2022).

### Kuwait

When *T. absoluta* was introduced in Kuwait, no registered insecticides controlled the pest. Hence, the insecticides recommended for the other pests, such as cypermethrin, were used for the control. Later, the diamides, chlorantraniliprole and flubendiamide were registered in 2015 and 2016, respectively. Between 2016 and 2017, eight *T. absoluta* field populations of Kuwait were subjected to baseline susceptible studies of chlorantraniliprole and flubendiamide. The population was susceptible against flubendiamide (RR: 2.75), a phthalic diamide insecticide group. Conversely, chlorantraniliprole (RR: 3.89), an anthranilic diamide showed a minor resistance level against *T. absoluta* (Jallow et al., 2019).

### Pakistan

In continuation with Brazil, Europe, Turkey and Kuwait, *T. absoluta* population of Pakistan had high level of resistance varying with RR from 43.13 to 59.88. When the population was selected up to 13 generations, the RR increased from 38.37 to 520.24 folds (Zang et al., 2022).

### India

The Indian population of *T. absoluta* evaluated during 2017-2018 against different group of insecticides such as diamides (chlorantraniliprole and flubendiamide), spinosyns (spinosad), oxadizines (indoxacarb), neonicotinoids (imidacloprid), and organophosphates (chlorpyriphos) had shown wide range of resistance level. Based on RR values, chlorantraniliprole, spinosad, flubendiamide, and chlorpyriphos was susceptible with maximum RR value of 2.21, 2.76, 2.21 and 1.72, respectively. Conversely, Indian population of *T. absoluta* had minor to low level of resistance against imidacloprid (RR: 6.6) and indoxacarb (RR: 7.72) (Kumar et al., 2020).

Similarly, between 2019 and 2020, different Indian field populations tested with indoxacarb, flubendiamide, cyantraniliprole, emamectin benzoate, spinetoram and Spinosad. In contrast to Kumar et al., 2020, indoxacarb and spinosad were susceptible with RR values 3 and 1.96, respectively. The *T. absoluta* population was found to be susceptible to spinetoram also. Whereas, minor level resistance has been reported for the insecticides, flubendiamide, cyantraniliprole, emamectin benzoate, and spinosad with RR values of 4.64, 3.90 and 3.45, respectively (Prasannakumar et al., 2021).

On an analytical view, maximum resistance level was shown by the insecticides indoxacarb, imidacloprid and flubendiamide. Surprisingly, within three years the indoxacarb which had minor to low level of resistance against tuta has become susceptible.

### South Africa

A significant degree of genetic similarity was also noted by Idriss (2019) in *T. absoluta* populations from Tanzania, Senegal, and Uganda. The Department of Agriculture, Forestry, and Fisheries (DAFF) authorized the emergency registration of insecticides to combat the *T. absoluta* infestation in South Africa. Indoxacarb, emamectin benzoate, spinetoram, and lufenuron are four insecticides IRAC recommends for controlling *T. absoluta* (DAFF, 2017). Among all the four insecticides tested against the African *T. absoluta* population exhibited that the insecticides indoxacarb, emamectin benzoate and spinetoram, was susceptible and only the lufenuron had a high level of resistance with a significant RR value of 47-fold increase (Hefer, 2021).

### Egypt

Methamidophos, methomyl, deltamethrin, spinosad, and imidacloprid were tested against the Egyptian tuta populations in the year 2010 to 2012. Out of all insecticides, deltamethrin (RR: 28.09 to 70.81) with sodium channel modulators as the mode of action had intermediate to high-level resistance. Other insecticides methamidophos, methomyl, spinosad, and imidacloprid had intermediate levels of resistance with maximum RR values 29.72, 32.53, 38.04 and 13.57, respectively (El-kady, 2012).

When an another tuta Egyptian population was evaluated in the year 2020 with insecticides belonging to a broader group of insecticides such as λ -cyhalothrin, chlorpyriphos, chlorantraniliprole, imidacloprid, emamectin benzoate, spinosad and indoxacarb had exhibited a varying level of resistance among which chlorantraniliprole (RR: 1.50) and indoxacarb (RR: 1.85) were susceptible while imidacloprid (RR: 3.63) showed minor level of resistance. In the order of increase, other insecticides emamectin benzoate (RR: 5.46), spinosad (RR: 6.25 to 23), chlorpyriphos (8.29 to 91.11), λ -cyhalothrin (RR: 10.94) had low, low to intermediate, low to high and intermediate levels of resistance, respectively (Mahmoud et al., 2021).

Overall, the Egyptian populations were exposed to varying insecticides with different mode of actions but the insecticides deltamethrin and chlorpyriphos had the maximum RR fold suggesting for the insecticide resistance management approaches.

## Overview of the global resistance status

Permethrin, cartap, abamectin, and methamidophos tested on Brazilian populations revealed that the cartap, a Nereistoxin analog with the mode of action against nicotinic acetylcholine receptor (nAChR) channel blockers (IRAC group 14), had the highest RR (21.9-fold) for Araguari population. Failure of cartap may be due to poor application methods. The low to moderate resistance developed for the widely used cartap might be due to a slower rate of resistance evolution in *T. absoluta*. However, increased use of abamectin, cartap, and permethrin by growers resulted in higher resistance to these chemicals. This result implied that the variance in susceptibility was primarily caused by local variations in pesticide use (Siqueira et al., 2000). As the Tuta gained resistance against certain chemicals, farmers started using diamides. The diamides of the IRAC group 28 act as the insect ryanodine receptor (RYR) modulator. With a 9.33-fold tolerance to anthranilic diamides, the Anapolis (ANP) population demonstrated the highest resistance, indicating that resistance did not have time to develop. As a result, Brazilian populations continued to exhibit the insecticide-induced natural response (Campos et al., 2014). The pyrethroids such as alpha-cypermethrin, deltamethrin, and permethrin were tested on a similar population where the Venda nova population had higher RR (10.8-fold) for alpha-cypermethrin with sodium channel modulator as mode of action (IRAC group 3A). *T. absoluta* populations in Brazil exhibited high levels of target site resistance for pyrethroid, which could be attributed to metabolic detoxification resistance (Silva et al., 2015). When abamectin, cartap, chlorfenapyr, indoxacarb, and metaflumizone were tested against the Brazilian populations, the Paulinia (PLP) population showed higher RR (21.2-fold) against metaflumizone, the IRAC group 22B insecticide that act as sodium channel blocker. It showed heterogeneity of susceptibility among *T. absoluta* populations (Silva et al., 2016a).

Among the European populations tested against chlorantraniliprole and indoxacarb, the Greece population (GR-IER3) showed the highest RR of 10-fold against the indoxacarb (IRAC group 22A) with sodium channel blocker as the mode of action. The resistance may be due to natural variation (Roditakis et al., 2013). A four-year survey and susceptibility study conducted by Roditakis et al. (2018) on the Italy, Greece and Spain populations against chlorantraniliprole, emamectin benzoate, spinosad, and indoxacarb showed higher RR (22,573) against chlorantraniliprole due to existence of detoxification and target site resistance mechanisms.

When the Kuwait population was tested against chlorantraniliprole and flubendiamide, the Wafra (WAF-3) population showed a higher RR (3.89-fold) for chlorantraniliprole. It suggested the presence of resistant alleles in the population (Jallow et al., 2019).

The Turkey populations were tested against abamectin, a glutamate-gated chloride channel (GluCl) allosteric modulator (IRAC group 6), where the Adana population showed a higher RR (3.03-fold). The differences in resistance levels indicated the presence of distinct selection pressures, such as using varying amounts of abamectin insecticide in various sites where *T. absoluta* samples were collected. Also, cytochrome P450 enzymes were revealed to play a significant role in developing abamectin resistance (Konuş, 2014). When the Turkey population was treated with chlorantraniliprole, metaflumizone, indoxacarb, and spinosad, the indoxacarb had a higher RR (8.02-fold) compared to other insecticides, and the population was found to have increased Glutathione S- transferase activity (Yalcin et al., 2015).

When indoxacarb was used as a test insecticide in Iran, the Ziba Shahr population had a higher RR (25.83-fold). Excessive usage of the same insecticide was the primary reason for the resistance (Taleh et al., 2023). Abamectin showed higher resistance against Shahre-e-Abrisham 1 population with RR value of 25.25. The primary enzymes glutathione S transferase and esterase were found to be highly involved in insecticide detoxification (Azizi and Khajehali, 2022).

Indian populations exposed to chlorantraniliprole, flubendiamide, spinosad, and indoxacarb showed higher RR for chlorantraniliprole (7.55-fold) and indoxacarb (7.72-fold). The existence of genetic polymorphisms induced natural variability that favored resistance development (Kumar et al., 2020). Among the Indian populations tested against indoxacarb, flubendiamide, emamectin benzoate, spinetoram, spinosad, and cyantraniliprole, flubendiamide showed higher RR (4.64-fold). It was presumed due to indiscriminate and frequent insecticide use (Prasannakumar et al., 2021).

Deltamethrin, methamidophos, and abamectin were tested against the Argentina population, showing a high RR (63.8-fold) for deltamethrin, a sodium channel modulator (IRAC group 3A). One possible explanation for the resistance to deltamethrin could be the intense selective pressure that pyrethroids impose on this population (Lietti et al., 2005). Lufenuron, a chemical not registered for use against *T. absoluta*, showed a 47-fold RR in the South African populations, whose effective control would be attained if applied at the registered dosage rate (Hefer, 2021).

Among the Pakistan population exposed to flubendiamide in 2018, 2019, and 2020, the Multan population in 2019 showed 59.88-fold resistance. These results implied that, in the laboratory, resistant alleles might have become more frequent as flubendiamide concentration was increased (Zang et al., 2022).

**Figure 2:**
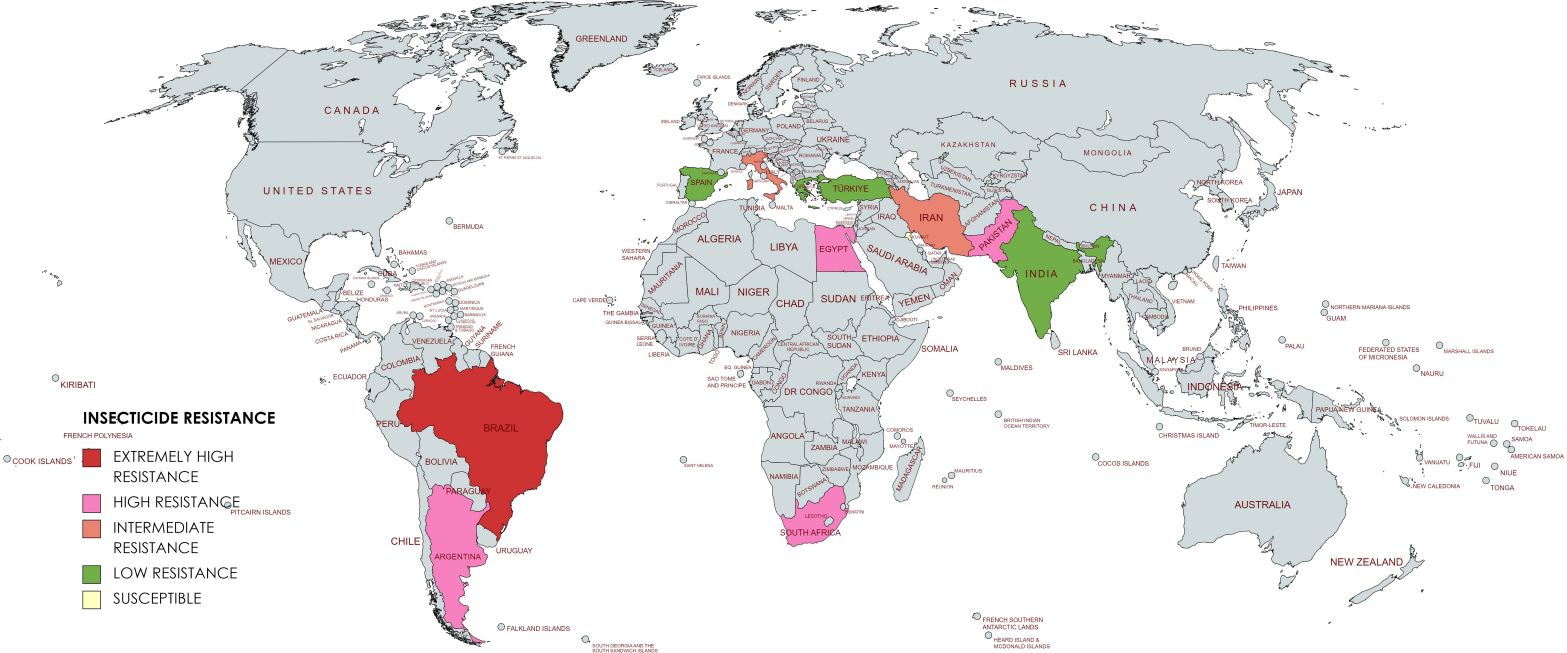
Global resistance status of *T. absoluta*.

Egyptian populations Damytta (DAM) population showed an RR of 70.81-fold for deltamethrin. The varying degrees of resistance could be attributed to variations in the pesticide application practices adopted across the several locations where the populations were collected (El-kady, 2012). Based on the above information the global resistance status has been depicted in **Figure 2**.

### Probable flow and facts for resistance

*T. absoluta* was first documented as attaining pest status in Brazil between September 1979 and October 1980, with the registration of pyrethroids in 1980 for their management. In 2000, the first case of resistance against permethrin was documented in the Brazilian *T. absoluta* population. Selection pressure and genetic diversity for the resistance mechanism among the populations were referred to as potential causes for resistance buildup. Permethrin and methamidaphos resistance were lowered, while cartap and abamectin were widely employed to control *T. absoluta* in Brazil. Later, target site resistance was frequent in *T. absoluta* Brazilian populations, and resistance became more robust due to metabolic detoxification capabilities.

*T. absoluta* was introduced into Southern Europe in 2006, and the pest permeated the whole continent by 2009, while insecticide resistance to many molecules emanated fast by 2010. The F1845Y and V1848I mutations in segment 6 of the sodium channel Domain IV were strongly associated with indoxacarb resistance. Abamectin-resistant *T. absoluta* exhibited an increased metabolic detoxification, and *T. absoluta* had evolved numerous mutations in GluCls, the target location of avermectins that dominated the populations. Spinosad resistance in *T. absoluta* had been associated with nAChR a6 subunit exon skipping, target site changes (G275E mutation in the nicotinic acetylcholine receptor a6 subunit), and metabolic resistance (increased cytochrome P450 activity). The presence of two new mutations (G4903V and I4746T) in the RyR transmembrane domain conferred resistance to diamide insecticides.

*T. absoluta* invaded Egypt in 2009, and insecticide resistance was reported in 2012 within a shorter span after introduction. The different degrees of resistance are most likely due to differences in how insecticides were applied in the various areas where the populations were collected. *T. absoluta* entered Kuwait in 2010, and by 2016, populations had developed insecticide resistance. It might be attributable to resistance alleles in the population that increased under selection pressure. *T. absoluta* was first detected in Iran in November 2010, and resistance had already been established by 2014. *T. absoluta* populations were resistant from the start of the invasion, and target site mutations and the impact of metabolic detoxification aided resistance development. Tuta was first imported to Argentina from Chile in April 1964, and insecticide resistance was discovered in 2000. Argentina’s most frequent insecticide resistance mechanism was metabolic detoxification for pyrethroids and abamectin. Following the invasion of Morocco in 2008, *T. absoluta* expanded throughout the whole African continent in eight years, reached South Africa in August 2016, and resistance developed in 2019. Dispersal might have delegated the geographic spread of insecticide resistance. Likewise, *T. absoluta* was introduced into Pakistan in 2016, and resistance emerged in 2018. The frequency of resistance alleles might have increased with increasing insecticide concentrations employed in plant protection. In India, this pest was found in Pune in October 2014, and insecticide resistance emerged in 2017; extensive usage of insecticides was recognized as the leading source of insecticide resistance buildup.

### Insecticide resistance management

IRM approach has two components: 1) reducing insecticide-induced selection pressure, and 2) avoiding insect-mediated resistance selection processes. The repeated use of the same chemical across generations results in insecticide resistance. As a result, lowering pest populations and optimizing insecticide use may reduce selection pressure. Integrated Pest Management (IPM), which includes cultural control, behavioral control (pheromone or color traps), biological control, and genetic control, can be implemented to reduce the need for insecticides, lowering the selection pressure on pest populations (Guedes and Picanço, 2012; Biondi et al., 2018). The convergence of cultural, behavioral, biological, and chemical control is the cornerstone of an efficient and long-lasting management strategy for *T. absoluta* (IRAC, 2014).

## Scouting and monitoring

Scouting is a regular field observation made during the crop cycle. The crop has to be scouted for *T. absoluta* symptoms, such as eggs, larvae, pupae or moths, and silvery tunnels on the leaves (Rwomushana et al., 2019). Monitoring determines the pest’s early existence and provides a population estimate. Insect traps, such as light traps, colored sticky traps, and pheromone traps, are used to monitor pest levels. Four kinds of pheromone traps are employed to monitor and mass trap *T. absoluta*. They are delta, light, water, and multi-lure traps. Placing sticky and pheromone traps in crucial areas like nurseries and fields at least two weeks before planting tomato crops helped in the practical decision-making of management measures (Rwomushana et al., 2019). 2–3 traps per hectare for field crops and 1 trap per 400m^2^ in greenhouses are used for monitoring, and 40-50 traps per hectare in fields and 20-25 traps per hectare in greenhouses (Rwomushana et al., 2019) are the recommended trap density for mass trapping.

### Cultural control

Cultural control is the process of altering the crop environment to (i) prevent the crop susceptibility stage from meeting the pest density peak, (ii) enhance crop growth conditions, or (iii) create an environment that is unfavorable to the pest (Rwomushana et al., 2019). Techniques for cultural control include i). Creation of proper conditions for growing healthy crops supplying required plant nutrients, thus increasing plants’ ability to withstand insect damage. *T. absoluta* takes a longer time to grow on fertilized soils. ii) Crop rotation with non-solanaceous crops: When rotated with beans, cabbage, maize, onions, and fodder grasses, tomato is manifested with low *T. absoluta*. If crop rotation is not followed, a minimum of 6 weeks should be allowed, from crop destruction to planting the next tomato crop, to prevent the spread of the pest. iii) Intercropping tomato with sesame increases the natural enemies of *T. absoluta*.

### Biological control

*Trichogramma pretiosum*, *Trichogramma acheae*, and *Trichogramma euproctidis* proved technically viable as biocontrol agents (up to 90% parasitism) in tomato greenhouses in Brazil, Spain, and Egypt, respectively. Parasitoid *Dolichogenidea gelechiidivoris* has an average parasitism of 70%. Two predatory mirid bugs, *Nesidiocoris tenuis*, and *Macrolophus pygmaeus*, feed on *T. absoluta* eggs and larvae. Entomopathogenic nematodes (EPNs), *Steinernema feltiae* produced high larval mortality (78-100%) (Mansour et al., 2018; Rwomushana et al., 2019).

Entomopathogenic fungi *Metarhizium anisopliae* infects and kills *T. absoluta* adults and larvae in their fourth instar, which lowers pupation and adult emergence. Different potent isolates of *M. anisophliae* such as ICIPE 18, ICIPE 20, and ICIPE 665, caused *T. absoluta* adult mortality of 95%, 87%, and 86%, respectively (Akutse et al., 2020).

### Botanicals

Botanicals are plant extracts that are applied topically or systemically to control pests. *T. absoluta* is controlled by using neem oil (Azadirachtin), an extract from neem (*Azadirachta indica*) seeds, as a contact insecticide (Kona et al., 2014).

Prevention of selection for resistance mechanisms and establishing cross-resistance is achieved by rotating pesticides. Using the pesticide mode of action (MoA) window method is appreciable to prevent cross-resistance. The Insecticide Resistance Action Committee (IRAC) MoA Classification Scheme will be an effective tool for choosing chemicals for a rotation scheme (Sparks and Nauen, 2015).

## Mode of action window approach for *T. absoluta*

Ensuring proper rotation of insecticides based on MoA is to refrain from treating successive generations of the target pest with insecticides in the same MoA group. Based on the minimal duration of a single generation of *T. absoluta*, a treatment window is established for 30 consecutive days. Applying insecticides from the same or different MoAs more than once within a specific window is possible. A second MoA window should be chosen for use in the next 30 days following the completion of the first 30-day MoA window if additional insecticide applications are required based on specified thresholds. In the same way, distinct MoAs for the next 30 days should be used in a third MoA frame. The plan aims to reduce resistance to a particular MoA group by prohibiting the reapplication of the same insecticide MoA group for at least 60 days following the closure of a window. It is a prudent precaution considering the possibility of a longer life cycle due to variations in temperature during the growing season. This scheme requires a minimum of three effective insecticide MoA groups, but ideally, more MoA groups should be included if locally registered and effective against *T. absoluta* (IRAC, 2014).

## Conclusion and future perspectives

*T. absoluta* is prevalent and has become a regular pest on tomatoes in more than 100 countries, damaging leaves, petioles, developing fruits, and other parts, challenging economical yields. The pest had created havoc among the growers and mirrored intensive pest suppression strategies, predominated by applying insecticides. Due to the extensive and repeated use of insecticides, the implied selection pressure ingressed the *T. absoluta* population to resist insecticides in more than 50 countries. The pest garnered attention for implementing insecticide resistance management (IRM) activities. The baseline susceptibility assessment of insecticides used for *T. absoluta* management is the initial step in determining a specific insecticide’s resistance status in the future. Overall, the review unveiled the different insecticides used to control *T. absoluta* and their corresponding resistance status in different countries. In the initial phases, *T. absoluta* took at least 20 years to develop resistance against insecticide molecules, but today, it takes little more than two years. The most prevalent resistance mechanism for rapid resistance development in *T. absoluta* was metabolic detoxification in conjunction with target-site mutation. Growers shall practice the application of chemical insecticides with management approaches like mating disruption and biological control to suppress populations. It may limit the frequency of resistance alleles and provide long-term *T. absoluta* control. Implementing effective IPM methods and mode of action window strategy is assumed to be the most effective way to delay the development of resistance to new insecticides in *T. absoluta*. Global assessment of baseline toxicity, identification of resistance pathways and molecular mechanisms, and the genetics of resistance may help prospect successful *T. absoluta* management. Implementing RNA interference (RNAi) strategies targeting primary resistance favoring molecular mechanisms at the genetic level may douse *T. absoluta* populations effectively.

## Author contributions

CB: Conceptualization, Methodology, Resources, Writing – original draft, Writing – review & editing. MM: Conceptualization, Methodology, Supervision, Writing – original draft, Writing – review & editing. VB: Writing – original draft, Writing – review & editing. SH: Writing – original draft, Writing – review & editing. KP: Writing – review & editing.

## References

[B1] Aboutalebian-Soureshjani A. Rafiee-Dastjerdi H. Naseri B. Hassanpour M. Khajehali J. (2023). Indoxacarb resistance in Iranian populations of *Tuta absoluta* (Lepidoptera: Gelechiidae): Cross-resistance, biochemical and molecular mechanisms. Pestic. Biochem. Physiol. 196, 105633. doi: 10.1016/j.pestbp.2023.105633 37945235

[B2] Akutse F. X. Subramanian S. Khamis F. M. Ekesi S. Mohamed S. A. (2020). Entomopathogenic fungus isolates for adult *Tuta absoluta* (Lepidoptera: Gelechiidae) management and their compatibility with *Tuta* pheromone. J. Appl. Entomol. 144, 777–787. doi: 10.1111/jen.12812

[B3] APRD. (2024). Arthropod pesticide resistance database Michigan state university. Available online at: https://www.pesticideresistance.org/ (Accessed January 01, 2024).

[B4] Arno J. Gabarra R. Molina P. Godfrey K. E. Zalom F. G. (2019). *Tuta absoluta* (Lepidoptera: Gelechiidae) success on common solanaceous species from California tomato production areas. Environ. Entomol. 48, 1394–1400. doi: 10.1093/ee/nvz109 31598654

[B5] Aslan B. Birgücü A. K. Uluişik S. Karaca I. (2022). Life table parameters of *Tuta absoluta* (Meyrick 1917) (Lepidoptera: Gelechiidae) on four wild tomato species. Turk. J. Entomol. 46, 175–186. doi: 10.16970/entoted.1016214

[B6] Azizi M. Khajehali J. (2022). Evaluation of resistance to abamectin in the populations of Tuta absoluta (Lepidoptera: gelechiidae), collected from Isfahan Province, Iran. J. Agric. Sci. Technol. 24, 379–391.

[B7] Ballal C. R. Gupta A. Mohan M. Lalitha Y. Verghese A. (2016). The new invasive pest *Tuta absoluta* (Meyrick)(Lepidoptera: Gelechiidae) in India and its natural enemies along with evaluation of Trichogrammatids for its biological control. Curr. Sci. 110, 2155–2159. doi: 10.18520/cs/v110/i11/2155-2159

[B8] Barros E. C. Bacci L. Picanco M. C. Martins J. C. Rosado J. F. Silva G. A. (2014). Physiological selectivity and activity reduction of insecticides by rainfall to predatory wasps of *Tuta absoluta* . J. Environ. Sci. Health - B. 50, 45–54. doi: 10.1080/03601234.2015.965621 25421627

[B9] Batalla-Carrera L. Morton A. García-del-Pino F. (2010). Efficacy of entomopathogenic nematodes against the tomato leafminer *Tuta absoluta* in laboratory and greenhouse conditions. Biocontrol 55, 523–530. doi: 10.1007/s10526-010-9284-z

[B10] Berxolli A. Shahini S. (2017). Population dynamic of tomato leaf miner, *Tuta absoluta* (Meyrick)(Lepidoptera: Gelechiidae). Alb. J. Agric. Sci., 85–89.

[B11] Biondi A. Guedes R. N. C. Wan F. H. Desneux N. (2018). Ecology, worldwide spread, and management of the invasive South American tomato pinworm, *Tuta absoluta*: past, present, and future. Ann. Rev. Entomol. 63, 239–258. doi: 10.1146/annurev-ento-031616-034933 28977774

[B12] Brévault T. Sylla S. Diatte M. Bernadas G. Diarra K. (2014). *Tuta absoluta* Meyrick (Lepidoptera: Gelechiidae): a new threat to tomato production in Sub-Saharan Africa. Afr. Entomol. 22, 441–444. doi: 10.4001/003.022.0202

[B13] Campos M. R. Rodrigues A. R. S. Silva W. M. Silva T. B.M. Silva V. R.F. Guedes R. N.C.. (2014). Spinosad and the tomato borer *Tuta absoluta*: a bioinsecticide, an invasive pest threat, and high insecticide resistance. PloS One 9, e103235. doi: 10.1371/journal.pone.0103235 25122089 PMC4133407

[B14] Campos M. R. Silva T. B. Silva W. M. Silva J. E. Siqueira A. (2015). Susceptibility of *Tuta absoluta* (Lepidoptera: Gelechiidae) Brazilian populations to ryanodine receptor modulators. Pest. Manage. Sci. 71, 537–544. doi: 10.1002/ps.3835 24863675

[B15] DAFF. (2017). Guideline for registered agrochemicals to control Tomato leafminer (*Tuta absoluta*) in South Africa. (South Africa: Department of Agriculture, Forestry and Fisheries).

[B16] Desneux N. Luna M. G. Guillemaud T. Urbaneja A. (2011). The invasive South American tomato pinworm, *Tuta absoluta*, continues to spread in Afro-Eurasia and beyond: the new threat to tomato world production. J. Pest. Sci. 84, 403–408. doi: 10.1007/s10340-011-0398-6

[B17] Desneux N. Wajnberg E. Wyckhuys K. A. Burgio G. Arpaia S. Narváez-Vasquez C. A.. (2010). Biological invasion of European tomato crops by *Tuta absoluta*: Ecology, geographic expansion and prospects for biological control. J. Pest. Sci. 83, 197–215. doi: 10.1007/s10340-010-0321-6

[B18] Douris V. Papapostolou K. M. Ilias A. Roditakis E. Kounadi S. Riga M.. (2017). Investigation of the contribution of RyR target-site mutations in diamide resistance by CRISPR/Cas9 genome modification in Drosophila. Insect. Biochem. Mol. Biol. 87, 127–135. doi: 10.1016/j.ibmb.2017.06.013 28669775

[B19] El-kady H. A. (2012). Insecticide resistance of tomato borer *Tuta absoluta* (meyrick) under Egyptian conditions. J. Plant Prot. Pathol. 3, 1089–1097. doi: 10.21608/jppp.2012.84397

[B20] EPPO. (2005). Data sheets on quarantine pests: *Tuta absoluta* . EPPO Bull. 35, 434–435.

[B21] EPPO. (2024). EPPO Global Database. Available online at: https://gd.eppo.int (Accessed 03 January, 2024).

[B22] Food and Agriculture Organization of the United Nations (FAO). (2021). FAOSTAT Statistical Database. Available online at: http://faostat.fao.org/faostat.

[B23] Gontijo P. C. Picanço M. C. Pereira E. J. G. Martins J. C. Chediak M. Guedes R. N. C. (2013). Spatial and temporal variation in the control failure likelihood of the tomato leaf miner, *Tuta absoluta* . Ann. Appl. Biol. 162, 50–59. doi: 10.1111/aab.12000

[B24] Grant C. Jacobson R. Ilias A. Berger M. Vasakis E. Bielza P.. (2019). The evolution of multiple-insecticide resistance in UK populations of tomato leafminer, *Tuta absoluta* . Pest. Manage. Sci. 75, 2079–2085. doi: 10.1002/ps.5381 30785238

[B25] Grant C. Singh K. S. Hayward A. Hunt B. J. Troczka B. J. Pym A.. (2023). Overexpression of the UDP-glycosyltransferase UGT34A23 confers resistance to the diamide insecticide chlorantraniliprole in the tomato leafminer, Tuta absoluta. Insect. Biochem. Mol. Biol. 159, 103983. doi: 10.1016/j.ibmb.2023.103983 37380137

[B26] Guedes R. N. C. Picanço M. C. (2012). The tomato borer *Tuta absoluta* in South America: pest status, management and insecticide resistance. EPPO Bull. 42, 211–216. doi: 10.1111/epp.2557

[B27] Guedes R. N. Siqueira H. A. (2012). The tomato borer *Tuta absoluta*: insecticide resistance and control failure. CABI Rev., 1–7. doi: 10.1079/PAVSNNR20127055

[B28] Hefer J. P. (2021). Host plant suitability and baseline susceptibility of *Tuta absoluta* (Lepidoptera: Gelechiidae) to insecticides in South Africa. South Africa: North-West University. Dissertation.

[B29] Idriss G. E. A. (2019). Bio-ecological studies of *Tuta absoluta* in Sudan. South Africa: North-West University. Dissertation.

[B30] Idriss G. E. A. Du Plessis H. Khamis F. M. Ekesi S. Tanga C. M. Mohamed S. A. (2020). Host range and effects of plant species on preference and fitness of *Tuta absoluta* (Lepidoptera: Gelechiidae). J. Econ. Entomol. 113, 1279–1289. doi: 10.1093/jee/toaa002 32016416

[B31] IRAC. (2014). *Tuta absoluta* - the tomato leafminer or tomato borer. Recommendations for Sustainable and Effective Resistance Management. Available online at: http://www.irac-online.org (Accessed 23 October, 2023).

[B32] IRAC. (2020). MoA Classification (Version 9.4). Available online at: http://www.irac-online.org (Accessed 07 November, 2023).

[B33] Jallow M. F. Dahab A. A. Albaho M. S. Devi V. Y. Awadh D. G. Thomas B. M. (2019). Baseline susceptibility and assessment of resistance risk to flubendiamide and chlorantraniliprole in *Tuta absoluta* (Lepidoptera: Gelechiidae) populations from Kuwait. Appl. Entomol. Zool. 54, 91–99. doi: 10.1007/s13355-018-0598-0

[B34] Jin T. Lin Y. Y. Jin Q. A. Wen H. B. Peng Z. Q. (2016). Population susceptibility to insecticides and the development of resistance in Bactrocera cucurbitae (Diptera: Tephritidae). J. Econ. Entomol. 109, 837–846. doi: 10.1093/jee/tov349 26668351

[B35] Kona N. E. M. Taha A. K. Mahmoud M. E. (2014). Effects of botanical extracts of Neem (*Azadirachta indica*) and jatropha (*Jatropha curcus*) on eggs and larvae of tomato leaf miner, *Tuta absoluta* (Meyrick)(Lepidoptera: Gelechiidae). PGCP 3, 41–46.

[B36] Konuş M. (2014). Analysing resistance of different *Tuta absoluta* (Meyrick)(Lepidoptera: Gelechiidae) strains to abamectin insecticide. Turk. J. Biochem. 39, 291. doi: 10.5505/tjb.2014.09327

[B37] Kumar J. S. Jayaraj J. Shanthi M. Theradimani M. Balasubramani V. Irulandi S.. (2020). Toxicity of insecticides to tomato pinworm, *Tuta absoluta* (Meyrick) populations from Tamil Nadu. Indian. J. Agric. Res. 54, 585–591. doi: 10.18805/IJARe.A-5443

[B38] Lietti M. M. Botto E. Alzogaray R. A. (2005). Insecticide resistance in argentine populations of *Tuta absoluta* (Meyrick)(Lepidoptera: Gelechiidae). Neotrop Entomol. 34, 113–119. doi: 10.1590/S1519-566X2005000100016

[B39] Mahmoud M. G. Hendawy M. A. Selem G. S. Salem R. E. (2021). Insecticides resistance spectrum in two field populations of *Tuta absoluta* (Meyrick) and λ-Cyhalothrin residues in tomato fruits. J. Plant Prot Pathol. 12, 757–763. doi: 10.21608/jppp.2021.214603

[B40] Mansour R. Brévault T. Chailleux A. Cherif A. Grissa-Lebdi K. Haddi K.. (2018). Occurrence, biology, natural enemies and management of *Tuta absoluta* in Africa. Entomol. Gen. 38, 83–112. doi: 10.1127/entomologia/2018/0749

[B41] Mohamed E. S. I. Mahmoud M. E. E. Elhaj M. A. M. Mohamed S. A. Ekesi S. (2015). Host plants record for tomato leaf miner *Tuta absoluta* (Meyrick) in Sudan. EPPO Bull. 45, 108–111. doi: 10.1111/epp.12178

[B42] Nauen R. (2006). Insecticide mode of action: return of the ryanodine receptor. Pest Manag Sci. 62, 690–692. doi: 10.1002/ps.1254 16770834

[B43] Nauen R. Slater R. Sparks T. C. Elbert A. Mccaffery A. (2019). “IRAC: insecticide resistance and mode-of-action classification of insecticides,” in Modern crop protection compounds. Eds. Jeschke P. Witschel M. Krämer W. Schirmer U. (Germany; Wiley-VCH Verlag GmbH & Co. KGaA), 995–1012. doi: 10.1002/9783527699261.ch28

[B44] Pandey P. Vadivelmurugan I. Bagavathiannan M. V. Senthil Kumar. M. (2017). Impact of combined abiotic and biotic stresses on plant growth and avenues for crop improvement by exploiting physio morphological traits. Front. Plant Sci. 8, 537–555. doi: 10.3389/fpls.2017.00537 28458674 PMC5394115

[B45] Peralta I. E. Spooner D. M. Knapp S. (2008). The taxonomy of tomatoes: a revision of wild tomatoes (Solanum sect. Lycopersicon) and their outgroup relatives (Solanum sect. Juglandifolia, sect. Lycopersicoides: Solanaceae). Syst. Bot. Monogr. 84, 1–186.

[B46] Prasannakumar N. R. Jyothi N. Saroja S. Kumar G. R. (2021). Relative toxicity and insecticide resistance of different field populations of tomato leaf miner, *Tuta absoluta* (Meyrick). Int. J. Trop. Insect. Sci. 41, 1397–1405. doi: 10.1007/s42690-020-00334-1

[B47] Reyes M. Rocha K. Alarcón L. Siegwart M. Sauphanor B. (2012). Metabolic mechanisms involved in the resistance of field populations of *Tuta absoluta* (Meyrick)(Lepidoptera: Gelechiidae) to spinosad. Pestic. Biochem. Physiol. 102, 45–50. doi: 10.1016/j.pestbp.2011.10.008

[B48] Roditakis E. Skarmoutsou C. Staurakaki M. del Rosario Martínez‐Aguirre M. García‐Vidal L. Bielza P.. (2013). Determination of baseline susceptibility of European populations of *Tuta absoluta* (Meyrick) to indoxacarb and chlorantraniliprole using a novel dip bioassay method. Pest. Manage. Sci. 69, 217–227. doi: 10.1002/ps.3404 23034903

[B49] Roditakis E. Steinbach D. Moritz G. Vasakis E. Stavrakaki M. Ilias A.. (2017). Ryanodine receptor point mutations confer diamide insecticide resistance in tomato leafminer, *Tuta absoluta* (Lepidoptera: Gelechiidae). Insect. Biochem. Mol. Biol. 80, 11–20. doi: 10.1016/j.ibmb.2016.11.003 27845250

[B50] Roditakis E. Vasakis E. García-Vidal L. del Rosario Martínez-Aguirre M. Rison J. L. Haxaire-Lutun M. O.. (2018). A four-year survey on insecticide resistance and likelihood of chemical control failure for tomato leaf miner *Tuta absoluta* in the European/Asian region. J. Pest. Sci. 91, 421–435. doi: 10.1007/s10340-017-0900-x

[B51] Roditakis E. Vasakis E. Grispou M. (2015). First report of *Tuta absoluta* resistance to diamide insecticides. J. Pest. Sci. 88, 9–16. doi: 10.1007/s10340-015-0643-5

[B52] Rwomushana I. Beale T. Chipabika G. Day R. Gonzalez-Moreno P. Lamontagne-Godwin J.. (2019). Tomato leafminer (*Tuta absoluta*): impacts and coping strategies for Africa. CABI Working Paper 12, 56. doi: 10.1079/CABICOMM-62-8100

[B53] Salama H. Fouda M. Ismail I. A. Ebada I. Shehata I. (2014). Life table parameters and fluctuations in the population density of the moth *Tuta absoluta* (Meyrick)-(Lepidoptera: Gelechiidae). Curr. Sci. Int. 3, 252–259.

[B54] Salazar E. R. Araya J. E. (2001). Tomato moth, *Tuta absoluta* (Meyrick) response to insecticides in Africa, Chile. Agr. Téc 61, 429–435.

[B55] Sawadogo W. M. Ahissou B. R. Somda I. Nacro S. Legrève A. Verheggen F. (2022). Identification of alternative hosts of the tomato leafminer *Tuta absoluta* (Meyrick 1917) (Lepidoptera: Gelechiidae) in West Africa. Afr Entomol. 30, 1–5. doi: 10.17159/2254-8854/2022/a12056

[B56] Silva J. E. Assis C. P. Ribeiro L. M. Siqueira H. A. (2016a). Field-evolved resistance and cross-resistance of Brazilian *Tuta absoluta* (Lepidoptera: Gelechiidae) populations to diamide insecticides. J. Econ. Entomol. 109, 2190–2195. doi: 10.1093/jee/tow161 27427509

[B57] Silva J. E. Ribeiro L. M. D. S. Vinasco N. Guedes R. N.C . Siqueira H. Á. A. (2019). Field-evolved resistance to chlorantraniliprole in the tomato pinworm Tuta absoluta: inheritance, cross-resistance profile, and metabolism. J. Pest. Sci. 92, 1421–1431. doi: 10.1007/s10340-018-1064-z

[B58] Silva J. E. Silva W. M. Silva T. B. M. Campos M. R. Filho A. B. E. Siqueira H. Á. A. (2021). High resistance to insect growth disruptors and control failure likelihood in Brazilian populations of the tomato pinworm Tuta absoluta. Phytoparasitica. 49, 689–701. doi: 10.1007/s12600-021-00895-y

[B59] Silva W. M. Berger M. Bass C. Balbino V. Q. Amaral M. H. Campos M. R.. (2015). Status of pyrethroid resistance and mechanisms in Brazilian populations of *Tuta absoluta* . Pestic. Biochem. Physiol. 122, 8–14. doi: 10.1016/j.pestbp.2015.01.011 26071801

[B60] Silva G. A. Picanço M. C. Bacci L. Crespo A. L. B. Rosado J. F. Guedes R. N. C. (2011). Control failure likelihood and spatial dependence of insecticide resistance in the tomato pinworm, *Tuta absoluta.* Pest. Manage. Sci. 67, 913–920. doi: 10.1002/ps.2131 21394881

[B61] Silva T. B. M. Silva W. M. Campos M. R. Silva J. E. Ribeiro L. M. S. Siqueira H. A. A. (2016b). Susceptibility levels of *Tuta absoluta* (Meyrick) (Lepidoptera: Gelechiidae) to minor classes of insecticides in Brazil. Crop Prot. 79, 80–86. doi: 10.1016/j.cropro.2015.10.012

[B62] Siqueira H. A. A. Guedes R. N. C. Fragoso D. D. B. Magalhaes L. C. (2001). Abamectin resistance and synergism in Brazilian populations of *Tuta absoluta* (Meyrick)(Lepidoptera: Gelechiidae). Int. J. Pest. Manage. 47, 247–251. doi: 10.1080/09670870110044634

[B63] Siqueira H. A. A. Guedes R. N. C. Picanco M. C. (2000). Cartap resistance and synergism in populations of *Tuta absoluta* (Lep., Gelechiidae). J. Appl. Entomol. 124, 233–238. doi: 10.1046/j.1439-0418.2000.00470.x

[B64] Souza J. C. Reis P. R. (1986). Controle da traça-do-tomateiroem Minas Gerais. Pesqui Agropec. Bras. 21, 343–354.

[B65] Sparks T. C. Nauen R. (2015). IRAC: Mode of action classification and insecticide resistance management. Pestic. Biochem. Physiol. 121, 122–128. doi: 10.1016/j.pestbp.2014.11.014 26047120

[B66] Sudo M. Takahashi D. Andow D. A. Suzuki Y. Yamanaka T. (2018). Optimal management strategy of insecticide resistance under various insect life histories: Heterogeneous timing of selection and interpatch dispersal. Evol. Appl. 11, 271–283. doi: 10.1111/eva.12550 29387161 PMC5775500

[B67] Taleh M. Garjan A. S. Rafiee-Dastjerdi H. Ebadollahi A. Noruzinia. G. (2023). Monitoring the susceptibility of different populations of tomato leaf miner, *Tuta absoluta* to indoxacarb and its combination with azadirachtin. J. Appl. Res. Plant Prot. 4, 131–139.

[B68] Tropea Garzia G. Siscaro G. Biondi A. Zappalà L. (2012). *Tuta absoluta*, an exotic invasive pest from South America now in the EPPO region: biology, distribution and damage. EPPO Bull. 42, 205–210. doi: 10.1111/epp.2556

[B69] Wakil W. Brust G. E. Perring T. (2018). Sustainable management of arthropod pests of tomato. J. Plant Prot. Res., 350–372.

[B70] Yalcin M. Mermer S. Kozaci L. D. Turgut C. (2015). Insecticide resistance in two populations of *Tuta absoluta* (Meyrick 1917)(Lepidoptera: Gelechiidae) from Turkey. Türki. Entomoloji. Derg. 39, 137–145. doi: 10.16970/ted.63047

[B71] Zang L. S. Akhtar Z. R. Ali A. Tariq K. Campos M. R. (2022). Flubendiamide resistance and its mode of inheritance in tomato pinworm *Tuta absoluta* (Meyrick)(Lepidoptera: gelechiidae). Insects 13, 1023. doi: 10.3390/insects13111023 36354846 PMC9693368

[B72] Zappalà L. Biondi A. Alma A. Al-Jboory I. J. Arnò J. Bayram A. (2013). Natural enemies of the South American moth, *Tuta absoluta*, in Europe, North Africa and Middle-East, and their potential use in pest control strategies. J. Pest. Sci. 86, 635–647. doi: 10.1007/s10340-013-0531-9

