# Supplementary material for: Baseline susceptibility of an A1 quarantine pest - the South American tomato pinworm Tuta absoluta (Lepidoptera: Gelechiidae) to insecticides: past incidents and future probabilities in line to implementing successful pest management
Source: Front Plant Sci. 2024 Aug 26;15:1404250. doi: 10.3389/fpls.2024.1404250 (PMC11404364; doi:10.3389/fpls.2024.1404250)
Supplement: Supplementary file 1 [file Table1.docx]

**Table S1. First report of *T. absoluta* in different countries using EPPO database**

| **Country** | **Place** | **First reported year** | **Main host** | **EPPO list*** |
| --- | --- | --- | --- | --- |
| Spain  (Southern Europe) | Castellón (Comunidad Valenciana) | 2006 | Tomato (*Lycopersicon esculentum),*  aubergine*(Solanum melongena),*potato*(S. tuberosum),*pepino*(S. muricatum*), and solanaceous weeds*(Datura stramonium, Lycium chilense,* and*S. nigrum*) | A1 |
|  | Mediterranean coast in the province of Valencia (Comunidad Valenciana) and Islas Baleares (Ibiza) | 2007 | Protected tomato crops | A1 |
| Algeria  (North Africa) | commune of Mazagran | 2008 | Protected tomato crops | A1 |
| Morocco | Bouareg in the region of Nador, North-Eastern Morocco | 2008 | Outdoor tomato crops (*Lycopersicon esculentum*) | A1 |
| France | Corse (near Ajaccio and Propriano) | October, 2008 | Tomato crops (*Lycopersicon* *esculentum*) | A1 |
|  | Provence-Alpes-Côte d’Azur (Var and Bouches-du-Rhône) | November/  December, 2008 |  | A1 |
| Italy | Province of Cosenza (Calabria) | Spring, 2008 | Glasshouse tomatoes (*Lycopersicon esculentum*) | A1 |
|  | Sardegna | Autumn, 2008 | Field and glasshouse tomatoes | A1 |
|  | Campania (Portici, Napoli) | Novembe,r 2008 | Experimental tomato glasshouse | A1 |
|  | Sicilia (Caltanissetta,Ragusa) | December, 2008 | Tomato glasshouses | A1 |
|  | Lazio  communes of Fondi, Terracina (both in the province of Latina), and Fiumicino (Roma) | 2009 | Glasshouse tomatoes | A1 |
|  | Abruzzo  municipality of San Salvo (Province of Chieti) |  | Field tomato crops (*Lycopersicon esculentum*) | A1 |
|  | Liguria  municipality of Albenga (Province of Savona) | 2009 | Glasshouse tomatoes (*L. esculentum* cv. ‘Cuore di Bue’) | A1 |
|  | Umbria  (Province of Perugia) |  | Pheromone traps located in tomato crops | A1 |
|  | Sicilia | 2009 | *Phaseolus vulgaris* | A1 |
| Tunisia | Akkouda (Sahel) | October, 2008 | Field tomato crops (*Lycopersicon esculentum*) | A1 |
| Netherlands | - | January, 2009 | Tomato Packing station | A1 |
|  |  | May, 2009 | Glasshouse of the same tomato packing station | A1 |
| United Kingdom | Essex, South East of England | March, 2009 | Imported Spanish tomato fruit at a packing station | A1 |
| Albania | Romanat (County of Durrës), Levan (County of Fier) and Rrogozhinë (County of Tirana) | 2009 | Tomato fields | A2 |
| Portugal | Algarve region (South of Portugal) | May, 2009 | Glasshouse producing tomato fruits (*Lycopersicon esculentum*) | A2 |
| Italy | Puglia  (Province of Lecce) | June, 2009 | Glasshouse producing tomato | A2 |
|  | Veneto  municipalities of Verona, Cavallino Treporti (Province of Venezia), and Lusia (Province of Rovigo) | July, 2009 | Glasshouse tomatoes | A2 |
| Malta | Dingli (South-West Malta) | April, 2009 | greenhouse | A2 |
| Switzerland | Geneva | July, 2009 | Glasshouse tomato-producing areas | A2 |
| Spain | Islas Canarias (Gran Canaria, Tenerife and Fuerteventura) | December, 2008 | Tomato-producing areas | A2 |
| Italy | Basilicata, Lombardia, and Molise | 2009 | Pheromone traps placed in tomato crops | A2 |
| Bulgaria | - | - | Territory | A2 |
| Cyprus | - | November, 2009 | Glasshouse tomato-producing areas | A2 |
| Germany | Baden-Württemberg | 2009 | Tomato crops, central markets, and packing stations | A2 |
| Italy | Piemonte region | 2009 | Glasshouse tomato crops | A2 |
| Israel | - | December, 2009 | - | A2 |
| Kosovo (YU) | Mamusha and Rahovec (county of Prizren) and Shtime (county of Ferizaj) | 2010 | Glasshouse areas | A2 |
| Hungary | Kiskunfélegyháza | 2010 | Field tomatoes | A2 |
| Guernsey | - | 2010 | Packing units and commercial glasshouses | A2 |
| Greece | Crete (Chania, Heraklion), Peloponnese (Achaea, Trifilia), and Western Greece (Preveza) | 2009 | Glasshouse and field tomato crops | A2 |
| Lithuania | Pagiriai, Vidmantai and NaujosiosKietaviškės | 2009 | - | A2 |
| Iraq | Rabia | Autumn, 2010 | Tomato crops | A2 |
| Trentino-Alto Adige region (IT), Italy | provinces of Trento and Bolzano | 2009 | Indoor and outdoor tomato crops | A2 |
| Mediterranean Basin | Bahrain, Kuwait,  Egypt, Jordan, Lebanon, Libya, Saudi Arabia, Sudan and Syria | 2009-2011 | Tomato and potato crops | A2 |
| Croatia | Turanj (Zadarska county) | 2009 | Hydroponic production of cherry tomatoes | A2 |
| Austria | Burgenland | 2010 | - | A2 |
| Russia | Krasnodar region (Southern Russia) | 2010 | Glasshouse tomatoes | A2 |
| Slovenia | Slovenia | 2009 | Glasshouse tomatoes | A2 |
| Panama | Río Sereno (Renacimiento district, Chiriquí province) | 2011 | Tomato crops | A2 |
| France | Aquitaine, Bretagne, Centre, Champagne-Ardenne, Corse, Ile-de-France, Languedoc-Roussillon, Midi-Pyrénées, Pays-de-la-Loire, Provence-Alpes-Côte d’Azur, Rhône-Alpes | 2011 | Tomato crops | A2 |
| Qatar | Al-Khor | March, 2011 | Glasshouse tomato plants | A2 |
| United Arab Emirates | - | July, 2012 | Aubergine (*Solanum lycopersicum, S. melongena*) grown in greenhouses | A2 |
| Yemen | Sana’a, Hudeidah, Lahj, Abyan, and Al-Baydah | January, 2013 | Tomato (*Solanum lycopersicum*) | A2 |
| Czech Republic | Prostějov (Olomouc region) | August, 2013 | Tomato crop (*Solanum lycopersicum*) | A2 |
| India | Pune (Maharashtra) | October, 2014 | Tomato (*Solanum lycopersicum*) plants grown under plastic tunnels and in the field. | A2 |
| Mayotte | Island of Mayotte | August, 2015 | Protected crops of tomato (*Solanum lycopersicum*) and aubergine (*S. melongen*a) | A2 |
| Nepal | Kathmandu | May, 2016 | Commercial tomato (*Solanum lycopersicum*) | A2 |
| South Africa | eastern border of the Mpumalanga province with Mozambique | August, 2016 | Pheromone traps | A2 |
| Uganda | Central Uganda | 2016 | Tomato (*Solanum lycopersicum*) | A2 |
| Zambia | Northern, Central, and Lusaka provinces | 2016 | Pheromone traps | A2 |
| Kyrgyzstan | Bishkek | October, 2016 | Tomato (*Solanum lycopersicum*) | A2 |
| Norway | Municipality of Klepp, Rogaland County | April, 2017 | Tomato glasshouse | A2 |
| Tajikistan | Khatlon and Republican Subordination regions | 2016 | Glasshouse and field tomato (*Solanum lycopersicum*) | A2 |
| Ghana | Berekusu (Eastern region of Ghana) | July, 2017 | Greenhouse company | A2 |
| China | Xinjiang province | August, 2017 | Tomato (*Solanum lycopersicum*) | A2 |

*In September 2004, the EPPO Council decided to add the pest *Tuta absoluta* to the EPPO A1 list. In September 2009, the EPPO Council approved the addition of the following pests to the EPPO A1 and A2 Lists of pests recommended for regulation.
