# Supplementary material for: Baseline susceptibility of an A1 quarantine pest - the South American tomato pinworm Tuta absoluta (Lepidoptera: Gelechiidae) to insecticides: past incidents and future probabilities in line to implementing successful pest management
Source: Front Plant Sci. 2024 Aug 26;15:1404250. doi: 10.3389/fpls.2024.1404250 (PMC11404364; doi:10.3389/fpls.2024.1404250)
Supplement: Supplementary file 2 [file Table2.docx]

| Supplementary Table S2. Additional information on worldwide baseline susceptibility studies in *Tuta absoluta* populations | | | | | | | | |
| --- | --- | --- | --- | --- | --- | --- | --- | --- |
| **Tuta population** | **Insecticide** | **Formulation/a.i** | **Brand** | **Manufacturer** | **LC 50** | **RR Fold increase** | **Year of Research** | **References** |
| **Argentina (2004)** | | | | | | | | |
| Castelar | Deltamethrin | 99% | - | Roussell-Uclaf, France | 0.35 μg a.i./larvae | 1.00 | 1993 | Lietti et al., 2005 |
| Rosario |  |  |  |  | 24.00 μg a.i./larvae | 68.38 |  |  |
| Bella vista |  |  |  |  | 24.00 μg a.i./larvae | 68.38 |  |  |
| Castelar | Methamidophos | 68% | - | Bayer, Argentina | 0.81 μg a.i./larvae | 1.00 | 1993 | Lietti et al., 2005 |
| Rosario |  |  |  |  | 0.65 μg a.i./larvae | 0.79 |  |  |
| Bella vista |  |  |  |  | 0.70 μg a.i./larvae | 0.86 |  |  |
| Castelar | Abamectin | 94% | - | Chemotecnica, Argentina | 0.16 μg a.i./larvae | 1.00 | 1993 | Lietti et al., 2005 |
| Rosario |  |  |  |  | 0.41 μg a.i./larvae | 2.48 |  |  |
| Bella vista |  |  |  |  | 0.57 μg a.i./larvae | 3.49 |  |  |
| **Brazil (1997-1998)** | | | | | | | | |
| Uberlandia | Abamectin | SP | - | Novartis, Biociencias, Sao Paulo | 0.97 μg a. i. /cm^2^ | 1.00 | 1998 | Siquiera et al., 2000 |
| Paulinia |  |  |  |  | 5.00 μg a. i. /cm^2^ | 5.16 | 1998 |  |
| Guiricema |  |  |  |  | 5.87 μg a. i. /cm^2^ | 6.04 | 1998 |  |
| Sao Joao da Barra |  |  |  |  | 5.96 μg a. i. /cm^2^ | 6.14 | 1997 |  |
| Viscosa |  |  |  |  | 6.76 μg a. i. /cm^2^ | 6.97 | 1997 |  |
| Lavras |  |  |  |  | 8.31 μg a. i. /cm^2^ | 8.57 | 1998 |  |
| Araguari |  |  |  |  | 9.09 μg a. i. /cm^2^ | 9.37 | 1998 |  |
| Paulinia | Cartap | SP | - | Iharabras, Sorocaba | 0.44 μg a. i. /cm^2^ | 1.00 | 1998 | Siquiera et al., 2000 |
| Guiricema |  |  |  |  | 0.97 μg a. i. /cm^2^ | 2.25 | 1998 |  |
| Lavras |  |  |  |  | 1.87 μg a. i. /cm^2^ | 4.22 | 1998 |  |
| Uberlandia |  |  |  |  | 4.57 μg a. i. /cm^2^ | 10.4 | 1998 |  |
| Viscosa |  |  |  |  | 4.81 μg a. i. /cm^2^ | 10.9 | 1997 |  |
| Sao Joao da Barra |  |  |  |  | 7.16 μg a. i. /cm^2^ | 16.2 | 1997 |  |
| Araguari |  |  |  |  | 9.68 μg a. i. /cm^2^ | 21.9 | 1998 |  |
| Sao Joao da Barra | Methamidaphos | SP | - | Bayer, Sao Paulo | 59.9 μg a. i. /cm^2^ | 1.00 | 1997 | Siquiera et al., 2000 |
| Viscosa |  |  |  |  | 62.3 μg a. i. /cm^2^ | 1.04 | 1997 |  |
| Guiricema |  |  |  |  | 79.3 μg a. i. /cm^2^ | 1.32 | 1998 |  |
| Lavras |  |  |  |  | 155 μg a. i. /cm^2^ | 2.59 | 1998 |  |
| Uberlandia |  |  |  |  | 191 μg a. i. /cm^2^ | 3.19 | 1998 |  |
| Araguari |  |  |  |  | 225 μg a. i. /cm^2^ | 3.76 | 1998 |  |
| Paulinia |  |  |  |  | 252 μg a. i. /cm^2^ | 4.22 | 1998 |  |
| Uberlandia | Permethrin | SP | - | Zeneca Agricola, Holambra | 47.8 μg a. i. /cm^2^ | 1.00 | 1998 | Siquiera et al., 2000 |
| Viscosa |  |  |  |  | 71.5 μg a. i. /cm^2^ | 1.5 | 1997 |  |
| Paulinia |  |  |  |  | 89.3 μg a. i. /cm^2^ | 1.87 | 1998 |  |
| Lavras |  |  |  |  | 142 μg a. i. /cm^2^ | 2.97 | 1998 |  |
| Sao Joao da Barra |  |  |  |  | 158 μg a. i. /cm^2^ | 3.31 | 1997 |  |
| Araguari |  |  |  |  | 187 μg a. i. /cm^2^ | 3.90 | 1998 |  |
| Guiricema |  |  |  |  | 316 μg a. i. /cm^2^ | 6.61 | 1998 |  |
| **Brazil (2010-2011)** | | | | | | | | |
| Guaraciaba do Norte (GBN) | Chlorantraniliprole | 200 g AI  L−1  SC | Prêmio® | DuPont, Paulínia, SP, Brazil | 3.17 mg L^−1^ | 1.00 | 2010 | Campos et al., 2014 |
| Venda Nova (VDN) |  |  |  |  | 5.70 mg L^−1^ | 1.79 | 2011 |  |
| Tianguá (TNG) |  |  |  |  | 6.92 mg L^−1^ | 2.18 | 2010 |  |
| Paulínia (PLN) |  |  |  |  | 7.05 mg L^−1^ | 2.22 | 2010 |  |
| Pelotas (PLT) |  |  |  |  | 9.05 mg L^−1^ | 2.85 | 2011 |  |
| Sumaré (SMR) |  |  |  |  | 10.23 mg L^−1^ | 3.22 | 2011 |  |
| Iraquara (IRQ) |  |  |  |  | 12.18 mg L^−1^ | 3.84 | 2011 |  |
| Anápolis (ANP) |  |  |  |  | 29.64 mg L^−1^ | 9.33 | 2011 |  |
| Guaraciaba do Norte (GBN) | Flubendiamide | 480 g AI L−1 SC | Belt® | Bayer S.A., Socorro, SP, Brazil | 94.0 μg a.i./L | 1.00 | 2010 | Campos et al., 2014 |
| Venda Nova (VDN) |  |  |  |  | 97.0 μg a.i./L | 1.03 | 2011 |  |
| Tianguá (TNG) |  |  |  |  | 99.0 μg a.i./L | 1.05 | 2010 |  |
| Paulínia (PLN) |  |  |  |  | 117.0 μg a.i./L | 1.24 | 2010 |  |
| Pelotas (PLT) |  |  |  |  | 123.0 μg a.i./L | 1.31 | 2011 |  |
| Sumaré (SMR) |  |  |  |  | 130.0 μg a.i./L | 1.39 | 2011 |  |
| Iraquara (IRQ) |  |  |  |  | 181.0 μg a.i./L | 1.93 | 2011 |  |
| Anápolis (ANP) |  |  |  |  | 230.0 μg a.i./L | 2.45 | 2011 |  |
| Guaraciaba do Norte (GBN) | Cyantraniliprole | 100 g AI L−1 OD | _ | DuPont, Paulínia, SP, Brazil | 8.61 μg a.i./L | 1.00 | 2010 | Campos et al., 2014 |
| Venda Nova (VDN) |  |  |  |  | 9.95 μg a.i./L | 1.15 | 2011 |  |
| Tianguá (TNG) |  |  |  |  | 16.17 μg a.i./L | 1.87 | 2010 |  |
| Paulínia (PLN) |  |  |  |  | 20.38 μg a.i./L | 2.37 | 2010 |  |
| Pelotas (PLT) |  |  |  |  | 20.60 μg a.i./L | 2.39 | 2011 |  |
| Sumaré (SMR) |  |  |  |  | 23.88 μg a.i./L | 2.77 | 2011 |  |
| Iraquara (IRQ) |  |  |  |  | 26.78 μg a.i./L | 3.11 | 2011 |  |
| Anápolis (ANP) |  |  |  |  | 28.95 μg a.i./L | 3.36 | 2011 |  |
| **Brazil (2010-2011)** | | | | | | | | |
| Iraquara (IRQ) | Abamectin | 18 CE | Vertimec | Syngenta, Brazil | 0.54 mg L^−1^ | - | 2011 | Silva et al., 2016a |
| Anápolis (ANP) |  |  |  |  | 0.81 mg L^−1^ | 1.5 | 2011 |  |
| Venda Nova (VDN) |  |  |  |  | 1.07 mg L^−1^ | 2.0 | 2011 |  |
| Guaraciaba do Norte (GBN) |  |  |  |  | 1.22 mg L^−1^ | 2.3 | 2010 |  |
| Sumaré (SMR) |  |  |  |  | 1.27 mg L^−1^ | 2.4 | 2011 |  |
| Pelotas (PLT) |  |  |  |  | 1.64 mg L^−1^ | 3.0 | 2011 |  |
| Paulínia (PLN) |  |  |  |  | 2.29 mg L^−1^ | 4.2 | 2010 |  |
| Tianguá (TNG) |  |  |  |  | 3.38 mg L^−1^ | 6.2 | 2010 |  |
| Pelotas (PLT) | Cartap | 500 SP | Cartape | Iharabras, Brazil | 93.1 mg L^−1^ | - | 2011 | Silva et al., 2016a |
| Paulínia (PLN) |  |  |  |  | 142.4 mg L^−1^ | 1.5 | 2010 |  |
| Iraquara (IRQ) |  |  |  |  | 173.7 mg L^−1^ | 1.9 | 2011 |  |
| Venda Nova (VDN) |  |  |  |  | 227.3 mg L^−1^ | 2.5 | 2011 |  |
| Sumaré (SMR) |  |  |  |  | 314.7 mg L^−1^ | 3.4 | 2011 |  |
| Tianguá (TNG) |  |  |  |  | 359.5 mg L^−1^ | 3.8 | 2010 |  |
| Guaraciaba do Norte (GBN) |  |  |  |  | 582.0 mg L^−1^ | 6.3 | 2010 |  |
| Anápolis (ANP) |  |  |  |  | 589.8 mg L^−1^ | 6.4 | 2011 |  |
| Pelotas (PLT) | Chlorfenapyr | 240 SC | Pirate | BASF, Brazil | 0.62 mg L^−1^ | - | 2011 | Silva et al., 2016a |
| Iraquara (IRQ) |  |  |  |  | 0.88 mg L^−1^ | 1.4 | 2011 |  |
| Sumaré (SMR) |  |  |  |  | 0.91 mg L^−1^ | 1.5 | 2011 |  |
| Tianguá (TNG) |  |  |  |  | 1.59 mg L^−1^ | 2.6 | 2010 |  |
| Venda Nova (VDN) |  |  |  |  | 1.65 mg L^−1^ | 2.7 | 2011 |  |
| Anápolis (ANP) |  |  |  |  | 1.80 mg L^−1^ | 2.9 | 2011 |  |
| Guaraciaba do Norte (GBN) |  |  |  |  | 2.59 mg L^−1^ | 4.2 | 2010 |  |
| Paulínia (PLN) |  |  |  |  | 2.83 mg L^−1^ | 4.6 | 2011 |  |
| Iraquara (IRQ) | Indoxacarb | 300 WG | Rumo | Dupont, Brazil | 0.86 mg L^−1^ | - | 2011 | Silva et al., 2016a |
| Tianguá (TNG) |  |  |  |  | 0.92 mg L^−1^ | 1.1 | 2010 |  |
| Paulínia (PLN) |  |  |  |  | 1.35 mg L^−1^ | 1.6 | 2010 |  |
| Guaraciaba do Norte (GBN) |  |  |  |  | 1.40 mg L^−1^ | 1.6 | 2010 |  |
| Sumaré (SMR) |  |  |  |  | 1.62 mg L^−1^ | 1.9 | 2011 |  |
| Anápolis (ANP) |  |  |  |  | 1.82 mg L^−1^ | 2.1 | 2011 |  |
| Venda Nova (VDN) |  |  |  |  | 2.26 mg L^−1^ | 2.6 | 2011 |  |
| Pelotas (PLT) |  |  |  |  | 2.89 mg L^−1^ | 3.3 | 20112 |  |
| Iraquara (IRQ) | Metaflumizone | - | - | BASF, Brazil | 0.35 mg L^−1^ | - | 2011 | Silva et al., 2016a |
| Sumaré (SMR) |  |  |  |  | 0.87 mg L^−1^ | 2.5 | 2011 |  |
| Pelotas (PLT) |  |  |  |  | 2.59 mg L^−1^ | 7.4 | 2011 |  |
| Anápolis (ANP) |  |  |  |  | 2.92 mg L^−1^ | 8.3 | 2011 |  |
| Tianguá (TNG) |  |  |  |  | 3.44 mg L^−1^ | 9.8 | 2010 |  |
| Venda Nova (VDN) |  |  |  |  | 4.60 mg L^−1^ | 13.1 | 2011 |  |
| Guaraciaba do Norte (GBN) |  |  |  |  | 7.25 mg L^−1^ | 20.7 | 2010 |  |
| Paulínia (PLN) |  |  |  |  | 7.44 mg L^−1^ | 21.2 | 2010 |  |
| **Brazil (2010-2015)** | | | | | | | | |
| America Dourada (AMD) | chlorantraniliprole | 200 gL^-1^ SP | Premio | DuPont, Brazil | 0.0044 mg L^−1^ | 1.00 | 2014 | Silva et al., 2016b |
| Bezerros (BZR) |  |  |  |  | 0.0049 mg L^−1^ | 1.12 | 2015 |  |
| Gameleira I (GML I) |  |  |  |  | 0.195 mg L^−1^ | 44.55 | 2014 |  |
| Gameleira II (GML II) |  |  |  |  | 1.55 mg L^−1^ | 355.76 | 2014 |  |
| Guaraciaba do Norte (GBN) |  |  |  |  | 2.29 mg L^−1^ | 524.85 | 2010 |  |
| Joao Dourado I  (JDR I) |  |  |  |  | 2.88 mg L^−1^ | 658.37 | 2014 |  |
| Joao Dourado II  (JDR II) |  |  |  |  | 4.65 mg L^−1^ | 1064 | 2014 |  |
| Lagoa Grande (LGD) |  |  |  |  | 92.43 mg L^−1^ | 21155 | 2014 |  |
| Pesqueira (PSQ) |  |  |  |  | 646.32 mg L^−1^ | 47928 | 2014 |  |
| Brasılia (BSL) |  |  |  |  | 1262.7 mg L^−1^ | 288995 | 2014 |  |
| Brasılia (BSL) | Cyantraniliprole | 100gL^-1^ CSO | Benevia | DuPont, Brazil | 0.015 mg L^−1^ | 1.00 | 2014 | Silva et al., 2016b |
| Guaraciaba do Norte (GBN) |  |  |  |  | 0.0157 mg L^−1^ | 1.03 | 2010 |  |
| Bezerros (BZR) |  |  |  |  | 1.20 mg L^−1^ | 78.34 | 2015 |  |
| Lagoa Grande (LGD) |  |  |  |  | 1.67 mg L^−1^ | 109.07 | 2014 |  |
| Joao Dourado II  (JDR II) |  |  |  |  | 2.25 mg L^−1^ | 147.03 | 2014 |  |
| Joao Dourado I  (JDR I) |  |  |  |  | 8.49 mg L^−1^ | 555.80 | 2014 |  |
| Gameleira II (GML II) |  |  |  |  | 28.93 mg L^−1^ | 1895 | 2014 |  |
| Gameleira I (GML I) |  |  |  |  | 90.59 mg L^−1^ | 5932 | 2014 |  |
| Pesqueira (PSQ) |  |  |  |  | 152.87 mg L^−1^ | 10010 | 2014 |  |
| America Dourada (AMD) |  |  |  |  | 281.3 mg L^−1^ | 18423 | 2014 |  |
| Guaraciaba do Norte (GBN) | Flubendiamide | 480gL^-1^ SC | Belt | Bayer, Brazil | 0.038 mg L^−1^ | 1.00 | 2010 | Silva et al., 2016b |
| Brasılia (BSL) |  |  |  |  | 0.30 mg L^−1^ | 7.95 | 2014 |  |
| Bezerros (BZR) |  |  |  |  | 0.41 mg L^−1^ | 11.03 | 2015 |  |
| Joao Dourado I  (JDR I) |  |  |  |  | 202.83 mg L^−1^ | 5405 | 2014 |  |
| Joao Dourado II  (JDR II) |  |  |  |  | 221.48 mg L^−1^ | 5901 | 2014 |  |
| Lagoa Grande (LGD) |  |  |  |  | 673.4 mg L^−1^ | 17943 | 2014 |  |
| Gameleira II (GML II) |  |  |  |  | 1045 mg L^−1^ | 27854 | 2014 |  |
| Pesqueira (PSQ) |  |  |  |  | 1398 mg L^−1^ | 37254 | 2014 |  |
| America Dourada (AMD) |  |  |  |  | 2178 mg L^−1^ | 58044 | 2014 |  |
| Gameleira I (GML I) |  |  |  |  | 3018 mg L^−1^ | 80413 | 2014 |  |
| **Europe (2009-2011)** | | | | | | | | |
| Greece (NAGREF) | Indoxacarb | 30 WG | Steward | DuPont, France | 1.8 and 17.9 mg L^−1^ | 10 | 2010–2011 | Roditakis et al., 2013 |
| Italy (UC) |  |  |  |  | 0.93 and 10.8 mg L^−1^ | 12 | 2009–2011 |  |
| Spain (UPCT) |  |  |  |  | 0.20 and 0.70 mg L^−1^ | 4 | 2010–2011 |  |
| Greece (NAGREF) | Chlorantraniliprole | 35 WG | Altacor | DuPont, France | 0.10 and 0.56 mg L^−1^ | 6 | 2009–2011 | Roditakis et al., 2013 |
| Italy (UC) |  |  |  |  | 0.23 and 1.34 mg L^−1^ | 6 | 2009–2011 |  |
| Spain (UPCT) |  |  |  |  | 0.04 and 0.24 mg L^−1^ | 6 | 2009–2011 |  |
| **Europe (2012-2016)** | | | | | | | | |
| GR-Lab | Chlorantraniliprole | 35 WG | Altacor | DuPont, France | 0.31 mg L^−1^ | - | 2012-2016 | Roditakis et al., 2018 |
| ES-Sus |  |  |  |  | 0.32 mg L^−1^ | - | 2012-2016 |  |
| Italy |  |  |  |  | 5.12 to 838 mg L^−1^ | 154 to 2704 | 2014-2016 |  |
| Greece |  |  |  |  | 0.14 to >1000 mg L^−1^ | 0.5 to >3200 | 2012-2016 |  |
| Spain |  |  |  |  | 0.12 to 1.45 mg L^−1^ | 1 to 8 | 2012-2015 |  |
| GR-Lab | Emamectin benzoate | 095 SG | Affirm | Syngenta, UK | 0.97 mg L^−1^ | - | 2012-2016 | Roditakis et al., 2018 |
| ES-Sus |  |  |  |  | 0.66 mg L^−1^ | - | 2012-2016 |  |
| Italy |  |  |  |  | 0.87 to 12.8 mg L^−1^ | 1 to 12 | 2014-2016 |  |
| Greece |  |  |  |  | 0.65 to 88 mg L^−1^ | 1 to 91 | 2012-2016 |  |
| Spain |  |  |  |  | 0.22 to 0.8 mg L^−1^ | 0.3 to 1 | 2012-2015 |  |
| GR-Lab | Spinosad | 480 SC | Laser | Dow, USA | 0.05 mg L^−1^ | - | 2012-2016 | Roditakis et al., 2018 |
| ES-Sus |  |  |  |  | 0.01 mg L^−1^ | - | 2012-2016 |  |
| Italy |  |  |  |  | 0.22 to 1.74 mg L^−1^ | 4 to 35 | 2014-2016 |  |
| Greece |  |  |  |  | 0.07 to 0.6 mg L^−1^ | 1 to 12 | 2012-2016 |  |
| Spain |  |  |  |  | 0.01 to 0.04 mg L^−1^ | 0.1 to 0.2 | 2012-2015 |  |
| GR-Lab | Indoxocarb | 30 WG | Steward | DuPont, France | 0.27 mg L^−1^ | - | 2012-2016 | Roditakis et al., 2018 |
| ES-Sus |  |  |  |  | 0.29 mg L^−1^ | - | 2012-2016 |  |
| Italy |  |  |  |  | 0.11 to 0.45 mg L^−1^ | 0.4 to 2 | 2014-2016 |  |
| Greece |  |  |  |  | 0.09 to 0.59 mg L^−1^ | 0.3 to 2 | 2012-2016 |  |
| Spain |  |  |  |  | 0.01 to 0.16 mg L^−1^ | 0.03 to 1 | 2012-2015 |  |
| **Turkey (2011-2012)** | | | | | | | | |
| Ankara | Abamectin | 1.8 CS | Vertis | Turkey, Baydar Tarim | 0.448 mg L^−1^ | 1.31 | 2011-2012 | Konus et al., 2014 |
| Antalya |  |  |  |  | 0.798 mg L^−1^ | 2.3 |  |  |
| Adana |  |  |  |  | 1.034 mg L^−1^ | 3.03 |  |  |
| **Turkey (2011-2012)** | | | | | | | | |
| Aydın | Chlorantraniliprole | 35 WG | Altacor | BASF | 15.35 mg L-1 | 1.84 | 2011 | Yalcin et al., 2015 |
| Urla |  |  |  |  | 8.36 mg L-1 | 1.00 | 2012 |  |
| Aydın | Metaflumizone | SC | Alverde | BASF | 2091.4 mg L-1 | 3.79 | 2011 | Yalcin et al., 2015 |
| Urla |  |  |  |  | 550.47 mg L-1 | 1.00 | 2012 |  |
| Aydın | Indoxacarb | 150 SC | Avaunt | BASF | 215.26 mg L-1 | 8.02 | 2011 | Yalcin et al., 2015 |
| Urla |  |  |  |  | 26.81 mg L-1 | 1.00 | 2012 |  |
| Aydın | Spinosad | 480 g/l SC | Laser | Dow Agrosciences | 0.7 mg L-1 | 6.40 | 2011 | Yalcin et al., 2015 |
| Urla |  |  |  |  | 0.11 mg L-1 | 1.00 | 2012 |  |
| **Iran (2017-2018)** | | | | | | | | |
| IUT | Abamectin | 1.8% EC | Vertimec | Syngenta, Switzerland | 5.67 | - | 2017-2018 | Azizi and Khajehali, 2022 |
| Shahre-e-Abrisham 2 |  |  |  |  | 10.92 | 1.92 |  |  |
| Kondelan |  |  |  |  | 13.77 | 2.42 |  |  |
| Mourche Khort |  |  |  |  | 24.27 | 4.28 |  |  |
| Hasseh |  |  |  |  | 25.52 | 4.50 |  |  |
| Ruran |  |  |  |  | 94.50 | 16.66 |  |  |
| Karchegan |  |  |  |  | 111.32 | 19.63 |  |  |
| Falavarjan |  |  |  |  | 114.04 | 20.11 |  |  |
|  |  |  |  |  | 143.18 | 25.25 |  |  |
| **Iran (2020-2021)** | | | | | | | | |
| Benoot-e Bala | Indoxacarb | 15% SC | - | Ariashimi, Iran | 12.28 mg L^−1^ | 2.19 | 2020-2021 | Taleh et al., 2023 |
| Ardabil |  |  |  |  | 23.67 mg L^−1^ | 4.22 |  |  |
| Safiabad |  |  |  |  | 47.76 mg L^−1^ | 8.52 |  |  |
| Parsabad Moghan |  |  |  |  | 48.64 mg L^−1^ | 8.68 |  |  |
| Mohammad Shahr |  |  |  |  | 13.08 mg L^−1^ | 13.08 |  |  |
| Ziba Shahr |  |  |  |  | 144.6 8 mg L^−1^ | 25.83 |  |  |
| GR-IER-16-6-S8 |  |  |  |  | 0.66 mg L^−1^ | 4.00 |  |  |
| **Kuwait (2016-2017)** | | | | | | | | |
| Abdally  (ABD 1) | Flubendiamide | 24 WG | Belt | Bayer AG, Germany | 0.05 mg L^−1^ | 1.25 | 2016 | Jallow et al., 2018 |
| Abdally  (ABD 2) |  |  |  |  | 0.05 mg L^−1^ | 1.25 | 2016 |  |
| Abdally  (ABD 3) |  |  |  |  | 0.06 mg L^−1^ | 1.5 | 2016 |  |
| Sulaibiya  (SUL 1) |  |  |  |  | 0.04 mg L^−1^ | 1.00 | 2016 |  |
| Sulaibiya  (SUL 2) |  |  |  |  | 0.09 mg L^−1^ | 2.25 | 2016 |  |
| Wafra  (WAF 1) |  |  |  |  | 0.08 mg L^−1^ | 2.00 | 2016 |  |
| Wafra  (WAF 2) |  |  |  |  | 0.11 mg L^−1^ | 2.75 | 2017 |  |
| Wafra  (WAF 3) |  |  |  |  | 0.09 mg L^−1^ | 2.25 | 2017 |  |
| Abdally  (ABD 1) | Chlorantraniliprole | 35 WG | Altacor | DuPont, France | 0.46 mg L^−1^ | 1.59 | 2016 | Jallow et al., 2018 |
| Abdally  (ABD 2) |  |  |  |  | 0.51 mg L^−1^ | 1.77 | 2016 |  |
| Abdally  (ABD 3) |  |  |  |  | 0.39 mg L^−1^ | 1.34 | 2016 |  |
| Sulaibiya  (SUL 1) |  |  |  |  | 0.47 mg L^−1^ | 1.62 | 2016 |  |
| Sulaibiya  (SUL 2) |  |  |  |  | 0.29 mg L^−1^ | 1.00 | 2016 |  |
| Wafra  (WAF 1) |  |  |  |  | 0.52 mg L^−1^ | 1.79 | 2016 |  |
| Wafra  (WAF 2) |  |  |  |  | 0.74 mg L^−1^ | 2.55 | 2017 |  |
| Wafra  (WAF 3) |  |  |  |  | 1.13 mg L^−1^ | 3.89 | 2017 |  |
| **Pakistan (2018 -2020)** | | | | | | | | |
| Lahore  Fluben-S | Flubendiamide | 480 SC | Belt | Bayer S.A., Socorro, SP, Brazil | 0.97 µg/mL | 1 | 2018 | Zang et al., 2022 |
| Fluben-R |  |  |  |  | 49.57 µg/mL | 51.10 |  |  |
| Faisalabad  Fluben-S |  |  |  |  | 1.17 µg/mL | 1 |  |  |
| Fluben-R |  |  |  |  | 52.35 µg/mL | 44.74 |  |  |
| Multan  Fluben-S |  |  |  |  | 1.16 µg/mL | 1 |  |  |
| Fluben-R |  |  |  |  | 54.38 µg/mL | 46.87 |  |  |
| Sahiwal  Fluben-S |  |  |  |  | 1.24 µg/mL | 1 |  |  |
| Fluben-R |  |  |  |  | 56.48 µg/mL | 45.54 |  |  |
| Lahore  Fluben-S | Flubendiamide | 480 SC | Belt | Bayer S.A., Socorro, SP, Brazil | 1.20 µg/mL | 1 | 2019 | Zang et al., 2022 |
| Fluben-R |  |  |  |  | 51.76 µg/mL | 43.13 |  |  |
| Faisalabad  Fluben-S |  |  |  |  | 0.92 µg/mL | 1 |  |  |
| Fluben-R |  |  |  |  | 51.49 µg/mL | 55.96 |  |  |
| Multan  Fluben-S |  |  |  |  | 0.92 µg/mL | 1 |  |  |
| Fluben-R |  |  |  |  | 55.09 µg/mL | 59.88 |  |  |
| Sahiwal  Fluben-S |  |  |  |  | 1.09 µg/mL | 1 |  |  |
| Fluben-R |  |  |  |  | 50.58 µg/mL | 46.40 |  |  |
| Lahore  Fluben-S | Flubendiamide | 480 SC | Belt | Bayer S.A., Socorro, SP, Brazil | 1.14 µg/mL | 1 | 2020 | Zang et al., 2022 |
| Fluben-R |  |  |  |  | 50.56 µg/mL | 44.35 |  |  |
| Faisalabad  Fluben-S |  |  |  |  | 1.15 µg/mL | 1 |  |  |
| Fluben-R |  |  |  |  | 50.69 µg/mL | 44.07 |  |  |
| Multan  Fluben-S |  |  |  |  | 1.06 µg/mL | 1 |  |  |
| Fluben-R |  |  |  |  | 52.67 µg/mL | 49.68 |  |  |
| Sahiwal  Fluben-S |  |  |  |  | 0.98 µg/mL | 1 |  |  |
| Fluben-R |  |  |  |  | 54.72 µg/mL | 55.83 |  |  |
| **India (2017 -2018)** | | | | | | | | |
| Madurai | Chlorantraniliprole | 18.5 SC | _ | DuPont | 0.607 mg L^−1^ | 1.33 | 2018 | Kumar et al., 2020 |
| Krishnagiri |  |  |  |  | 0.371 mg L^−1^ | 2.21 | 2018 |  |
| Coimbatore |  |  |  |  | 0.608 mg L^−1^ | 1.07 | 2018 |  |
| Theni |  |  |  |  | 2.056 mg L^−1^ | 1.54 | 2017 |  |
| Dindigul |  |  |  |  | 0.312 mg L^−1^ | 1.51 | 2018 |  |
| Lab population |  |  |  |  | 0.272 mg L^−1^ | - | - |  |
| Madurai | Spinosad | 45 SC | _ | Dow AgroSciences | 0.813 mg L^−1^ | 2.47 | 2018 | Kumar et al., 2020 |
| Krishnagiri |  |  |  |  | 0.668 mg L^−1^ | 2.03 | 2018 |  |
| Coimbatore |  |  |  |  | 0.571 mg L^−1^ | 1.74 | 2018 |  |
| Theni |  |  |  |  | 0.905 mg L^−1^ | 2.76 | 2017 |  |
| Dindigul |  |  |  |  | 0.635 mg L^−1^ | 1.94 | 2018 |  |
| Lab population |  |  |  |  | 0.328 mg L^−1^ | - | - |  |
| Madurai | Flubendiamide | 480 SC | _ | Bayer | 1.354 mg L^−1^ | 1.33 | 2018 | Kumar et al., 2020 |
| Krishnagiri |  |  |  |  | 2.258 mg L^−1^ | 2.21 | 2018 |  |
| Coimbatore |  |  |  |  | 1.097 mg L^−1^ | 1.07 | 2018 |  |
| Theni |  |  |  |  | 1.566 mg L^−1^ | 1.54 | 2017 |  |
| Dindigul |  |  |  |  | 1.541 mg L^−1^ | 1.51 | 2018 |  |
| Lab population |  |  |  |  | 1.018 mg L^−1^ | - | - |  |
| Madurai | Imidacloprid | 17.8 SL | _ | Bayer | 5.430 mg L^−1^ | 5.5 | 2018 | Kumar et al., 2020 |
| Krishnagiri |  |  |  |  | 6.520 mg L^−1^ | 6.6 | 2018 |  |
| Coimbatore |  |  |  |  | 3.534 mg L^−1^ | 3.6 | 2018 |  |
| Theni |  |  |  |  | 4.654 mg L^−1^ | 4.7 | 2017 |  |
| Dindigul |  |  |  |  | 3.033 mg L^−1^ | 3.1 | 2018 |  |
| Lab population |  |  |  |  | 0.989 mg L^−1^ | - | - |  |
| Madurai | Chlorpyriphos | 20 EC | _ | Dow AgroSciences | 1911.98 mg L^−1^ | 1.23 | 2018 | Kumar et al., 2020 |
| Krishnagiri |  |  |  |  | 1041.81 mg L^−1^ | 1.07 | 2018 |  |
| Coimbatore |  |  |  |  | 1548.06 mg L^−1^ | 1.6 | 2018 |  |
| Theni |  |  |  |  | 1656.55 mg L^−1^ | 1.71 | 2017 |  |
| Dindigul |  |  |  |  | 1771.90 mg L^−1^ | 1.72 | 2018 |  |
| Lab population |  |  |  |  | 967.32 mg L^−1^ | - | - |  |
| Madurai | Indoxacarb | 14.5 SC | _ | DuPont | 2.918 mg L^−1^ | 3.53 | 2018 | Kumar et al., 2020 |
| Krishnagiri |  |  |  |  | 4.595 mg L^−1^ | 5.6 | 2018 |  |
| Coimbatore |  |  |  |  | 6.385 mg L^−1^ | 7.72 | 2018 |  |
| Theni |  |  |  |  | 5.439 mg L^−1^ | 6.6 | 2017 |  |
| Dindigul |  |  |  |  | 3.361 mg L^−1^ | 4.1 | 2018 |  |
| Lab population |  |  |  |  | 0.821 mg L^−1^ | - | - |  |
| **India (2019 -2020)** | | | | | | | | |
| Susceptible population | Indoxacarb | 14.5 SC | - | Syngenta | 11.036 mg/L^-1^ | - | 2019 - 2020 | Prasannakumar et al., 2020 |
| Bangalore |  |  |  |  | 11.779 mg/L^-1^ | 1.06 |  |  |
| Kolar |  |  |  |  | 22.85 mg/L^-1^ | 2.07 |  |  |
| Madurai |  |  |  |  | 10.887 mg/L^-1^ | 0.986 |  |  |
| Salem |  |  |  |  | 12.779 mg/L^-1^ | 1.44 |  |  |
| Anantapur |  |  |  |  | 33.216 mg/L^-1^ | 3.00 |  |  |
| Susceptible population | Flubendiamide | 39.35 SC | - | Bayer crop Science AG, Germany | 6.969 mg/L^-1^ | - | 2019 - 2020 | Prasannakumar et al., 2020 |
| Bangalore |  |  |  |  | 9.408 mg/L^-1^ | 1.34 |  |  |
| Kolar |  |  |  |  | 5.104 mg/L^-1^ | 0.732 |  |  |
| Madurai |  |  |  |  | 6.0915 mg/L^-1^ | 0.874 |  |  |
| Salem |  |  |  |  | 9.408 mg/L^-1^ | 1.052 |  |  |
| Anantapur |  |  |  |  | 32.343 mg/L^-1^ | 4.64 |  |  |
| Susceptible population | Emamectin benzoate | 5 SG | - | Syngenta | 7.598 mg/L^-1^ | - | 2019 - 2020 | Prasannakumar et al., 2020 |
| Bangalore |  |  |  |  | 8.534 mg/L^-1^ | 1.01 |  |  |
| Kolar |  |  |  |  | 8.427 mg/L^-1^ | 1.077 |  |  |
| Madurai |  |  |  |  | 5.640 mg/L^-1^ | 0.742 |  |  |
| Salem |  |  |  |  | 8.143 mg/L^-1^ | 1.07 |  |  |
| Anantapur |  |  |  |  | 29.270 mg/L^-1^ | 3.45 |  |  |
| Susceptible population | Spinosad | 45 SC | - | Dow Agrosciences | 5.595 mg/L^-1^ | - | 2019 - 2020 | Prasannakumar et al., 2020 |
| Bangalore |  |  |  |  | 6.6689 mg/L^-1^ | 1.19 |  |  |
| Kolar |  |  |  |  | 4.844 mg/L^-1^ | 0.865 |  |  |
| Madurai |  |  |  |  | 4.706 mg/L^-1^ | 0.841 |  |  |
| Salem |  |  |  |  | 6.236 mg/L^-1^ | 1.12 |  |  |
| Anantapur |  |  |  |  | 25.618 mg/L^-1^ | 1.96 |  |  |
| Susceptible population | Spinetoram | 11.7 SC |  | Dow Agrosciences | 11.886 mg/L^-1^ | - | 2019 - 2020 | Prasannakumar et al., 2020 |
| Bangalore |  |  |  |  | 13.034 mg/L^-1^ | 1.09 |  |  |
| Kolar |  |  |  |  | 12.071 mg/L^-1^ | 1.031 |  |  |
| Madurai |  |  |  |  | 12.556 mg/L^-1^ | 1.051 |  |  |
| Salem |  |  |  |  | 13.560 mg/L^-1^ | 1.11 |  |  |
| Anantapur |  |  |  |  | 4.608 mg/L^-1^ | 2.13 |  |  |
| Susceptible population | Cyantraniliprole | 10.25 SC |  | DuPont, France | 9.026 mg/L^-1^ | - | 2019 - 2020 | Prasannakumar et al., 2020 |
| Bangalore |  |  |  |  | 10.001 mg/L^-1^ | 1 |  |  |
| Kolar |  |  |  |  | 20.22 mg/L^-1^ | 2.24 |  |  |
| Madurai |  |  |  |  | 10.963 mg/L^-1^ | 1.231 |  |  |
| Salem |  |  |  |  | 13.256 mg/L^-1^ | 1.445 |  |  |
| Anantapur |  |  |  |  | 29.495 mg/L^-1^ | 3.20 |  |  |
| **South Africa (2019)** | | | | | | | | |
| Mareetsane | Emamectin benzoate | - | Proclaim | Syngenta | 0.05 μg a.i./larvae | 2.5 | 2019 | Hefer, 2021 |
| Polokwane |  |  |  |  | 0.02 μg a.i./larvae |  |  |  |
| Swartwater |  |  |  |  | 0.02 μg a.i./larvae |  |  |  |
| Mareetsane | Spinetoram | - | Delegate | Dow AgroSciences | 0.04 μg a.i./larvae | 2 | 2019 | Hefer, 2021 |
| Polokwane |  |  |  |  | 0.02 μg a.i./larvae |  |  |  |
| Swartwater |  |  |  |  | 0.03 μg a.i./larvae |  |  |  |
| Mareetsane | Indoxacarb | 30 WG | Steward | DuPont | 1.96 μg a.i./larvae | 1.77 | 2019 | Hefer, 2021 |
| Polokwane |  |  |  |  | 2.92 μg a.i./larvae |  |  |  |
| Swartwater |  |  |  |  | 3.47 μg a.i./larvae |  |  |  |
| Mareetsane | Lufenuron | - | Sorba | Syngenta | 0.18 μg a.i./larvae | 47 | 2019 | Hefer, 2021 |
| Polokwane |  |  |  |  | 0.47 μg a.i./larvae |  |  |  |
| Swartwater |  |  |  |  | 0.01 μg a.i./larvae |  |  |  |
| **Egypt (2010-2012)** | | | | | | | | |
| Susceptible  (SUS) | Methamidophos | 40% | Tamaron | - | 2.9  μg a.i./larvae | - | 2010 | El-kady, 2012 |
| Marsa Matrouh  (MAR) |  |  |  |  | 30.8  μg a.i./larvae | 10.62 | 2012 |  |
| Behera  (BEH) |  |  |  |  | 33.2  μg a.i./larvae | 11.45 | 2012 |  |
| Kafer  (KAF) |  |  |  |  | 39.3  μg a.i./larvae | 13.55 | 2012 |  |
| Damytta  (DAM) |  |  |  |  | 86.2  μg a.i./larvae | 29.72 | 2012 |  |
| Susceptible  (SUS) | Methomyl | 90% SP | Lannate | - | 4.58  μg a.i./larvae | - | 2010 | El-kady, 2012 |
| Marsa Matrouh  (MAR) |  |  |  |  | 49.3  μg a.i./larvae | 10.76 | 2012 |  |
| Behera  (BEH) |  |  |  |  | 132.2  μg a.i./larvae | 28.86 | 2012 |  |
| Kafer  (KAF) |  |  |  |  | 110.6  μg a.i./larvae | 24.15 | 2012 |  |
| Damytta  (DAM) |  |  |  |  | 149  μg a.i./larvae | 32.53 | 2012 |  |
| Susceptible  (SUS) | Deltamethrin | 25 g/l EC | Decis | - | 1.1  μg a.i./larvae | - | 2010 | El-kady, 2012 |
| Marsa Matrouh  (MAR) |  |  |  |  | 30.9 μg a.i./larvae | 28.09 | 2012 |  |
| Behera  (BEH) |  |  |  |  | 43.1  μg a.i./larvae | 39.18 | 2012 |  |
| Kafer  (KAF) |  |  |  |  | 42.4  μg a.i./larvae | 38.54 | 2012 |  |
| Damytta  (DAM) |  |  |  |  | 77.9  μg a.i./larvae | 70.81 | 2012 |  |
| Susceptible  (SUS) | Spinosad | 24% SC | Spintor | - | 0.24 μg a.i./larvae | - | 2010 | El-kady, 2012 |
| Marsa Matrouh  (MAR) |  |  |  |  | 4.1 μg a.i./larvae | 17.08 | 2012 |  |
| Behera  (BEH) |  |  |  |  | 7.67 μg a.i./larvae | 31.96 | 2012 |  |
| Kafer  (KAF) |  |  |  |  | 4.4 μg a.i./larvae | 18.33 | 2012 |  |
| Damytta  (DAM) |  |  |  |  | 9.13 μg a.i./larvae | 38.04 | 2012 |  |
| Susceptible  (SUS) | Imidacloprid | 200 g/l SL | Confidor | - | 0.14 μg a.i./larvae | - | 2010 | El-kady, 2012 |
| Marsa Matrouh  (MAR) |  |  |  |  | 1.9 μg a.i./larvae | 13.57 | 2012 |  |
| Behera  (BEH) |  |  |  |  | 1.7 μg a.i./larvae | 12.14 | 2012 |  |
| Kafer  (KAF) |  |  |  |  | 2.09 μg a.i./larvae | 14.92 | 2012 |  |
| Damytta  (DAM) |  |  |  |  | 1.9 μg a.i./larvae | 13.57 | 2012 |  |
| **Egypt (2020)** | | | | | | | | |
| El- Salhia | λ-cyhalothrin | 5% EC | - | El- Help manufacture | 1003.78 μg/ml | 10.94 | 2020 | Mahmoud et al., 2021 |
| Abo Kabeer |  |  |  |  | 173.17 μg/ml | 1.89 |  |  |
| Lab strain |  |  |  |  | 91.73 μg/ml | - |  |  |
| El- Salhia | Chlorpyrifos | 48% EC | - | El- Help manufacture | 3108.62 μg/ml | 91.11 | 2020 | Mahmoud et al., 2021 |
| Abo Kabeer |  |  |  |  | 282.82 μg/ml | 8.29 |  |  |
| Lab strain |  |  |  |  | 34.12 μg/ml | - |  |  |
| El- Salhia | Chlorantraniliprole | 20% SC | Coragen | Dupont Company | 29.48 μg/ml | 1.50 | 2020 | Mahmoud et al., 2021 |
| Abo Kabeer |  |  |  |  | 3.97 μg/ml | 0.20 |  |  |
| Lab strain |  |  |  |  | 19.63 μg/ml | - |  |  |
| El- Salhia | Imidacloprid | 24% WP | - | El- Help manufacture | 1319.16 μg/ml | 3.63 | 2020 | Mahmoud et al., 2021 |
| Abo Kabeer |  |  |  |  | 492.96 μg/ml | 1.36 |  |  |
| Lab strain |  |  |  |  | 363.05 μg/ml | - |  |  |
| El- Salhia | Emmamectin benzoate | 5% SG | Proclaim | Syngenta | 1.73 μg/ml | 5.41 | 2020 | Mahmoud et al., 2021 |
| Abo Kabeer |  |  |  |  | 0.18 μg/ml | 0.56 |  |  |
| Lab strain |  |  |  |  | 0.32 μg/ml | - |  |  |
| El- Salhia | Spinosad | 24% SC | Tracer | Dow agrociences | 0.92 μg/ml | 23.00 | 2020 | Mahmoud et al., 2021 |
| Abo Kabeer |  |  |  |  | 0.25 μg/ml | 6.25 |  |  |
| Lab strain |  |  |  |  | 0.04 μg/ml | - |  |  |
| El- Salhia | Indoxacarb | 15% SC | Advantage | Montajat Pharmaceutical company | 0.85 μg/ml | 1.85 | 2020 | Mahmoud et al., 2021 |
| Abo Kabeer |  |  |  |  | 0.15 μg/ml | 0.33 |  |  |
| Lab strain |  |  |  |  | 0.46 μg/ml | - |  |  |

CS- Capsule suspension

CSO- Concentrated suspension in oil

CE/ES- Emulsifiable concentrate

OD- Oil dispersible

SG- Soluble granules

SL- Soluble liquid

SP- Soluble powder

SC- Soluble concentrate

WG- Wettable granules

WP- Wettable powder
