# Supplementary material for: Baseline susceptibility of an A1 quarantine pest - the South American tomato pinworm Tuta absoluta (Lepidoptera: Gelechiidae) to insecticides: past incidents and future probabilities in line to implementing successful pest management
Source: Front Plant Sci. 2024 Aug 26;15:1404250. doi: 10.3389/fpls.2024.1404250 (PMC11404364; doi:10.3389/fpls.2024.1404250)
Supplement: Supplementary file 3 [file Table3.docx]

**Table S3. Baseline susceptibility of various insecticides of *T. absoluta* populations belonging to different countries/continents**

| **Tuta population** | **Insecticide/IRAC group** | **Insecticide class** | **MOA** | **Resistance level** | **Mechanism of resistance** | **References** |  |  |  |
| --- | --- | --- | --- | --- | --- | --- | --- | --- | --- |
| **Argentina (2004)** | | | | | | | |  |  |
| Castelar  Rosario  Bella vista | Deltamethrin  (Group 3A) | Pyrethroids | Sodium channel modulators | High resistance | Metabolic resistance  Target site alteration | Lietti et al., 2005 |  |  |  |
| Castelar  Rosario  Bella vista | Methamidophos  (Group 1B) | Organophosphates | Acetylcholinesterase (AChE) inhibitors | Susceptible | Metabolic resistance | Lietti et al., 2005 |  |  |  |
| Castelar  Rosario  Bella vista | Abamectin  (Group 6) | Avermectins | Glutamate-gated chloride channel (GluCl) allosteric modulators | Minor resistance | Metabolic resistance | Lietti et al., 2005 |  |  |  |
| **Brazilian (1997-1998)** | | | | | | | |  |  |
| Uberlandia  Paulinia  Guiricema  Sao Joao da Barra  Viscosa  Lavras  Araguari | Abamectin  (Group 6) | Avermectins | Glutamate-gated chloride channel (GluCl) allosteric modulators | Low-level | Metabolic resistance | Siquiera et al., 2000 |  |  |  |
| Uberlandia  Paulinia  Guiricema  Sao Joao da Barra  Viscosa  Lavras  Araguari | Cartap  (Group 14) | Nereistoxin analogues | Nicotinic acetylcholine receptor (nAChR) channel blockers | Minor to intermediate level | Metabolic resistance | Siquiera et al., 2000 |  |  |  |
| Uberlandia  Paulinia  Guiricema  Sao Joao da Barra  Viscosa  Lavras  Araguari | Methamidophos  (Group 1B) | Organophosphates | Acetylcholinesterase (AChE) inhibitors | Minor level | Metabolic resistance | Siquiera et al., 2000 |  |  |  |
| Uberlandia  Viscosa  Paulinia  Lavras  Sao Joao da Barra  Araguari  Guiricema | Permethrin  (Group 3A) | Pyrethroids | Sodium channel modulators | Minor to low-level | Metabolic resistance  Target site alteration | Siquiera et al., 2000 |  |  |  |
| **Brazilian (2010-2011)** | | | | | | | |  |  |
| Guaraciaba do Norte (GBN)  Venda Nova (VDN)  Tianguá (TNG)  Paulínia (PLN)  Pelotas (PLT)  Sumaré (SMR)  Iraquara (IRQ)  Anápolis (ANP) | Chlorantraniliprole  (Group 25) | Diamides | Ryanodine receptor modulators | Minor to low level | Metabolic resistance  Target site alteration | Campos et al., 2014 |  |  |  |
| Guaraciaba do Norte (GBN)  Venda Nova (VDN)  Tianguá (TNG)  Paulínia (PLN)  Pelotas (PLT)  Sumaré (SMR)  Iraquara (IRQ)  Anápolis (ANP) | Cyantraniliprole  (Group 25) | Diamides | Ryanodine receptor modulators | Minor level | Metabolic resistance  Target site alteration | Campos et al., 2014 |  |  |  |
| Guaraciaba do Norte (GBN)  Venda Nova (VDN)  Tianguá (TNG)  Paulínia (PLN)  Pelotas (PLT)  Sumaré (SMR)  Iraquara (IRQ)  Anápolis (ANP) | Flubendiamide  (Group 25) | Diamides | Ryanodine receptor modulators | Susceptible | Metabolic resistance  Target site alteration | Campos et al., 2014 |  |  |  |
| **Brazilian (2010-2011)** | | | | | | | |  |  |
| Guaraciaba do Norte (GBN)  Venda Nova (VDN)  Tianguá (TNG)  Paulínia (PLN)  Pelotas (PLT)  Sumaré (SMR)  Iraquara (IRQ)  Anápolis (ANP) | Abamectin  (Group 6) | Avermectins | Glutamate-gated chloride channel (GluCl) allosteric modulators | Minor to low level | Metabolic resistance | Silva et al., 2016a |  |  |  |
| Guaraciaba do Norte (GBN)  Venda Nova (VDN)  Tianguá (TNG)  Paulínia (PLN)  Pelotas (PLT)  Sumaré (SMR)  Iraquara (IRQ)  Anápolis (ANP) | Cartap  (Group 14) | Nereisoxin analogues | Nicotinic acetylcholine receptor (nAChR) channel blockers | Minor to low level | Metabolic resistance | Silva et al., 2016a |  |  |  |
| Guaraciaba do Norte (GBN)  Venda Nova (VDN)  Tianguá (TNG)  Paulínia (PLN)  Pelotas (PLT)  Sumaré (SMR)  Iraquara (IRQ)  Anápolis (ANP) | Chlorfenapyr  (Group 13) | Pyrroles | Uncouplers of oxidative phosphorylation via disruption of the proton gradient | Minor level | Metabolic resistance | Silva et al., 2016a |  |  |  |
| Guaraciaba do Norte (GBN)  Venda Nova (VDN)  Tianguá (TNG)  Paulínia (PLN)  Pelotas (PLT)  Sumaré (SMR)  Iraquara (IRQ)  Anápolis (ANP) | Indoxacarb  (Group 22A) | Oxadizines | Voltage-dependent sodium channel blockers | Minor level | Metabolic resistance  Target site alteration | Silva et al., 2016a |  |  |  |
| Guaraciaba do Norte (GBN)  Venda Nova (VDN)  Tianguá (TNG)  Paulínia (PLN)  Pelotas (PLT)  Sumaré (SMR)  Iraquara (IRQ)  Anápolis (ANP) | Metaflumizone  (Group 22A) | Oxadizines | Voltage-dependent sodium channel blockers | Intermediate level | Metabolic resistance | Silva et al., 2016a |  |  |  |
| **Brazilian (2010-2015)** | | | | | | | |  |  |
| America Dourada (AMD)  Bezerros (BZR)  Brasılia (BSL)  Gameleira I (GML I)  Gameleira II (GML II)  Guaraciaba do Norte (GBN)  Joao Dourado I (JDR I)  Joao Dourado II (JDR II)  Lagoa Grande (LGD)  Pesqueira (PSQ) | Chlorantraniliprole  (Group 25) | Diamides | Ryanodine receptor modulators | High to extremely high | Metabolic resistance  Target site alteration | Silva et al., 2016b |  |  |  |
| America Dourada (AMD)  Bezerros (BZR)  Brasılia (BSL)  Gameleira I (GML I)  Gameleira II (GML II)  Guaraciaba do Norte (GBN)  Joao Dourado I (JDR I)  Joao Dourado II (JDR II)  Lagoa Grande (LGD)  Pesqueira (PSQ) | Cyantraniliprole  (Group 25) | Diamides | Ryanodine receptor modulators | High to extremely high | Metabolic resistance | Silva et al., 2016b |  |  |  |
| America Dourada (AMD)  Bezerros (BZR)  Brasılia (BSL)  Gameleira I (GML I)  Gameleira II (GML II)  Guaraciaba do Norte (GBN)  Joao Dourado I (JDR I)  Joao Dourado II (JDR II)  Lagoa Grande (LGD)  Pesqueira (PSQ) | Flubendiamide  (Group 25) | Diamides | Ryanodine receptor modulators | Low to extremely high | Metabolic resistance  Target site alteration | Silva et al., 2016b |  |  |  |
| **Europe (2009-2011)** | | | | | | | |  |  |
| Greece (NAGREF)  Italy (UC)  Spain (UPCT) | Indoxacarb  (Group 22A) | Oxadizines | Voltage-dependent sodium channel blockers | Minor to low-level | Metabolic resistance  Target site alteration | Roditakis et al., 2013 |  |  |  |
| Greece (NAGREF)  Italy (UC)  Spain (UPCT) | Chlorantraniliprole  (Group 25) | Diamides | Ryanodine receptor modulators | Minor level | Metabolic resistance  Target site alteration | Roditakis et al., 2013 |  |  |  |
| **Europe (2012-2016)** | | | | | | | |  |  |
| Italy  Greece  Spain | Chlorantraniliprole  (Group 25) | Diamides | Ryanodine receptor modulators | Minor to extremely high level | Metabolic resistance  Target site alteration | Roditakis et al., 2018 |  |  |  |
| Italy  Greece  Spain | Emamectin benzoate  (Group 6) | Oxadizines | Glutamate-gated chloride channel (GluCl) allosteric modulators | Minor to high level | Metabolic resistance | Roditakis et al., 2018 |  |  |  |
| Italy  Greece  Spain | Spinosad  (Group 5) | Spinosyns | Nicotinic acetylcholine receptor (nAChR) allosteric modulators – Site I | Minor to intermediate level | Metabolic resistance  Target site alteration | Roditakis et al., 2018 |  |  |  |
| Italy  Greece  Spain | Indoxacarb  (Group 22A) | Oxadizines | Voltage-dependent sodium channel blockers | Minor to intermediate level | Metabolic resistance  Target site alteration | Roditakis et al., 2018 |  |  |  |
| **Turkey (2011-2012)** | | | | | | | |  |  |
| Ankara  Antalya  Adana | Abamectin  (Group 6) | Avermectins | Glutamate-gated chloride channel (GluCl) allosteric modulators | Susceptible | Metabolic resistance | Konus et al., 2014 |  |  |  |
| **Turkey (2011-2012)** | | | | | | | |  |  |
| Aydın | Chlorantraniliprole  (Group 25) | Diamides | Ryanodine receptor modulators | Susceptible | Metabolic resistance  Target site alteration | Yalcin et al., 2015 |  |  |  |
| Aydın | Metaflumizone  (Group 22B) | Oxadizines | Voltage -dependent sodium channel blockers | Minor level | Metabolic resistance | Yalcin et al., 2015 |  |  |  |
| Aydın | Indoxacarb  (Group 22B) | Oxadizines | Voltage -dependent sodium channel blockers | Low level | Metabolic resistance  Target site alteration | Yalcin et al., 2015 |  |  |  |
| Aydın | Spinosad  (Group 5) | Spinosyns | Nicotinic acetylcholine receptor (nAChR) allosteric modulators – Site I | Low level | Metabolic resistance  Target site alteration | Yalcin et al., 2015 |  |  |  |
| **Iran (2017-2018)** | | | | | | |  |  |  |
| IUT  Shahre-e-Abrisham 2  Kondelan  Mourche Khort  Hasseh  Ruran  Karchegan  Falavarjan  Shahre-e-Abrisham 1 | Abamectin  (Group 6) | Avermectins | Glutamate-gated chloride channel (GluCl) allosteric modulators | Minor to intermediate level | Metabolic resistance | Azizi and Khajehali, 2022 |  |  |  |
| **Iran (2020-2021)** | | | | | | | |  |  |
| Benoot-e Bala  Ardabil  Safiabad  Parsabad Moghan  Mohammad Shahr  Ziba Shahr | Indoxacarb  (Group 22 A) | Oxadizines | Voltage -dependent sodium channel blockers | Minor to intermediate level | Metabolic resistance | Taleh et al., 2023 |  |  |  |
| **Kuwait (2016-2017)** | | | | | | | |  |  |
| Abdally  (ABD 1)  Abdally  (ABD 2)  Abdally  (ABD 3)  Sulaibiya  (SUL 1)  Sulaibiya  (SUL 2)  Wafra  (WAF 1)  Wafra  (WAF 2)  Wafra  (WAF 3) | Flubendiamide  (Group 25) | Diamides | Ryanodine receptor modulators | Susceptible | Metabolic resistance  Target site alteration | Jallow et al., 2018 |  |  |  |
| Abdally  (ABD 1)  Abdally  (ABD 2)  Abdally  (ABD 3)  Sulaibiya  (SUL 1)  Sulaibiya  (SUL 2)  Wafra  (WAF 1)  Wafra  (WAF 2)  Wafra  (WAF 3) | Chlorantraniliprole  (Group 25) | Diamides | Ryanodine receptor modulators | Minor level | Metabolic resistance  Target site alteration | Jallow et al., 2018 |  |  |  |
| **Pakistan (2018-2020)** | | | | | | | |  |  |
| Lahore  Faisalabad  Multan  Sahiwal | Flubendiamide  (Group 25) | Diamides | Ryanodine receptor modulators | High level | Metabolic resistance  Target site alteration | Zang et al., 2022 |  |  |  |
| **India (2017-2018)** | | | | | | | |  |  |
| Madurai  Krishnagiri  Coimbatore  Theni  Dindigul | Chlorantraniliprole  (Group 25) | Diamides | Ryanodine receptor modulators | Susceptible | Metabolic resistance  Target site alteration | Kumar et al., 2020 |  |  |  |
| Madurai  Krishnagiri  Coimbatore  Theni  Dindigul | Spinosad  (Group 5) | Spinosyns | Nicotinic acetylcholine receptor (nAChR) allosteric modulators – Site I | Susceptible | Metabolic resistance  Target site alteration | Kumar et al., 2020 |  |  |  |
| Madurai  Krishnagiri  Coimbatore  Theni  Dindigul | Flubendiamide  (Group 25) | Diamides | Ryanodine receptor modulators | Susceptible | Metabolic resistance  Target site alteration | Kumar et al., 2020 |  |  |  |
| Madurai  Krishnagiri  Coimbatore  Theni  Dindigul | Imidacloprid  (Group 4A) | Neonicotinoids | Nicotinic acetylcholine receptor (nAChR) competitive modulators | Minor to low level | Metabolic resistance | Kumar et al., 2020 |  |  |  |
| Madurai  Krishnagiri  Coimbatore  Theni  Dindigul | Chlorpyriphos  (Group 1B) | Organophosphates | Acetylcholinesterase (AChE) inhibitors | Susceptible | Metabolic resistance | Kumar et al., 2020 |  |  |  |
| Madurai  Krishnagiri  Coimbatore  Theni  Dindigul | Indoxacarb  (Group 22A) | Oxadizines | Voltage-dependent sodium channel blockers | Minor to low low-level | Metabolic resistance  Target site alteration | Kumar et al., 2020 |  |  |  |
| **India (2019-2020)** | | | | | | | |  |  |
| Bangalore  Kolar  Madurai  Salem  Anantapur | Indoxacarb  (Group 22A) | Oxadizines | Voltage-dependent sodium channel blockers | Susceptible | Metabolic resistance  Target site alteration | Prasannakumar et al., 2020 |  |  |  |
| Bangalore  Kolar  Madurai  Salem  Anantapur | Flubendiamide  (Group 25) | Diamides | Ryanodine receptor modulators | Minor level | Metabolic resistance  Target site alteration | Prasannakumar et al., 2020 |  |  |  |
| Bangalore  Kolar  Madurai  Salem  Anantapur | Emamectin benzoate  (Group 6) | Avermectins | Glutamate -gated chloride channel (GluCl) allosteric modulators | Minor level | Metabolic resistance | Prasannakumar et al., 2020 |  |  |  |
| Bangalore  Kolar  Madurai  Salem  Anantapur | Spinosad  (Group 5) | Spinosyns | Nicotinic acetylcholine receptor (nAChR) allosteric modulators – Site I | Minor level | Metabolic resistance  Target site alteration | Prasannakumar et al., 2020 |  |  |  |
| Bangalore  Kolar  Madurai  Salem  Anantapur | Spinetoram  (Group 5) | Spinosyns | Nicotinic acetylcholine receptor (nAChR) allosteric modulators - site I | Susceptible | Metabolic resistance | Prasannakumar et al., 2020 |  |  |  |
| Bangalore  Kolar  Madurai  Salem  Anantapur | Cyantraniliprole  (Group 25) | Diamides | Ryanodine receptor modulators | Minor level | Metabolic resistance | Prasannakumar et al., 2020 |  |  |  |
| **South Africa (2019)** | | | | | | | |  |  |
| Mareetsane  Polokwane  Swartwater | Emamectin benzoate  (Group 6) | Avermectins | Glutamate -gated chloride channel (GluCl) allosteric modulators | Susceptible | Metabolic resistance | Hefer, 2021 |  |  |  |
| Mareetsane  Polokwane  Swartwater | Spinetoram  (Group 5) | Spinosyns | Nicotinic acetylcholine receptor (nAChR) allosteric modulators - site I | Susceptible | Metabolic resistance | Hefer, 2021 |  |  |  |
| Mareetsane  Polokwane  Swartwater | Indoxacarb  (Group 22A) | Oxadizines | Voltage-dependent sodium channel blockers | Susceptible | Metabolic resistance  Target site alteration | Hefer, 2021 |  |  |  |
| Mareetsane  Polokwane  Swartwater | Lufenuron  (Group 15) | Benzoylureas | Inhibitors of chitin biosynthesis affecting CHS1 | High level | Metabolic resistance | Hefer, 2021 |  |  |  |
| **Egypt (2010-2012)** | | | | | | | |  |  |
| Marsa Matrouh (MAR)  Behera (BEH)  Kafer (KAF)  Damytta (DAM) | Methamidophos  (Group 1A) | Organophosphates | Acetylcholinesterase (AChE) inhibitors | Intermediate level | Metabolic resistance | El -kady, 2012 |  |  |  |
| Marsa Matrouh (MAR)  Behera (BEH)  Kafer (KAF)  Damytta (DAM) | Methomyl  (Group 1A) | Carbamates | Acetylcholinesterase (AChE) inhibitors | Intermediate level | Metabolic resistance | El -kady, 2012 |  |  |  |
| Marsa Matrouh (MAR)  Behera (BEH)  Kafer (KAF)  Damytta (DAM) | Deltamethrin  (Group 3A) | Pyrethroids | Sodium channel modulators | Intermediate to high level | Metabolic resistance | El -kady, 2012 |  |  |  |
| Marsa Matrouh (MAR)  Behera (BEH)  Kafer (KAF)  Damytta (DAM) | Spinosad  (Group 5) | Spinosyns | Nicotinic acetylcholine receptor (nAChR) allosteric modulators – Site I | Intermediate level | Metabolic resistance  Target site alteration | El -kady, 2012 |  |  |  |
| Marsa Matrouh (MAR)  Behera (BEH)  Kafer (KAF)  Damytta (DAM) | Imidacloprid  (Group 4A) | Neonicotinoids | Nicotinic acetylcholine receptor (nAChR) competitive modulators | Intermediate level | Metabolic resistance | El -kady, 2012 |  |  |  |
| **Egypt (2020)** | | | | | | | |  |  |
| El- Salhia  Abo Kabeer | λ -cyhalothrin  (Group 3A) | Pyrethroids | Sodium channel modulators | Intermediate level | Metabolic resistance | Mahmoud et al., 2021 |  |  |  |
| El- Salhia  Abo Kabeer | Chlorpyriphos  (Group 1B) | Organophosphates | Acetylcholinesterase (AChE) inhibitors | Low to high | Metabolic resistance | Mahmoud et al., 2021 |  |  |  |
| El- Salhia  Abo Kabeer | Chlorantraniliprole  (Group 25) | Diamides | Ryanodine receptor modulators | Susceptible | Metabolic resistance  Target site alteration | Mahmoud et al., 2021 |  |  |  |
| El- Salhia  Abo Kabeer | Imidacloprid  (Group 4A) | Neonicotinoids | Nicotinic acetylcholine receptor (nAChR) competitive modulators | Minor level | Metabolic resistance | Mahmoud et al., 2021 |  |  |  |
| El- Salhia  Abo Kabeer | Emamectin benzoate  (Group 6) | Avermectins | Glutamate -gated chloride channel (GluCl) allosteric modulators | Low-level | Metabolic resistance | Mahmoud et al., 2021 |  |  |  |
| El- Salhia  Abo Kabeer | Spinosad  (Group 5) | Spinosyns | Nicotinic acetylcholine receptor (nAChR) allosteric modulators – Site I | Low to intermediate | Metabolic resistance  Target site alteration | Mahmoud et al., 2021 |  |  |  |
| El- Salhia  Abo Kabeer | Indoxacarb  (Group 22A) | Oxadizines | Voltage-dependent sodium channel blockers | Susceptible | Metabolic resistance  Target site alteration | Mahmoud et al., 2021 |  |  |  |
